# Systematic review on modification to the *ad-hoc* on-demand distance vector routing discovery mechanics

**DOI:** 10.7717/peerj-cs.1079

**Published:** 2022-09-05

**Authors:** Ibrahim Alameri, Jitka Komarkova, Tawfik Al-Hadhrami, Ahmad Lotfi

**Affiliations:** 1 Computer Science, Nottingham Trent University, Nottingham, United Kingdom; 2 University of Pardubice, Pardubice, Czech Republic; 3 Jabir ibn Hayyan Medical University, Najaf, Iraq

**Keywords:** Routing protocol, Mechanics, *ad-hoc*, AODV, Wireless mesh network

## Abstract

Mobile *ad-hoc* networks (MANETs) and wireless mesh networks (WMNs) are used in a variety of research areas, including the military, industry, healthcare, agriculture, the Internet of Things (IoT), transportation, and smart cities. The swift advancement in MANET technology is the driving force behind this rising adoption rate. Routing over MANET is a critical problem due to the dynamic nature of the link qualities, even when nodes are static. A key challenge in MANETs is the need for an efficient routing protocol that establishes a route according to certain performance metrics related to the link quality. The routing protocols utilised by the nodes in WMNs and MANETs are distinct. Nodes in both types of networks exchange data packets through the routing protocols. For this highly mobile network, the *ad-hoc* On-Demand Distance Vector (AODV) routing protocol has been suggested as a possible solution. Recent years have attracted researchers’ attention to AODV since it is a routing technique for *ad-hoc* networks that prevents looping. The architecture of this routing protocol considers several factors, including the mobility of nodes, the failure of connection links, and the loss of packets. In this systematic review, one of the key focuses is bringing attention to the classic AODV, which was developed after discussing the recent development of several versions of AODV. The AODV routing protocol performs a path strength check to generate a more reliable and secure route between the source and destination nodes. In AODV, investigations demonstrate advances in both the format protocol approach and the network simulation-2 (NS-2), and these improvements were made in the same scenario used to revitalise AODV. It has been discovered that the AODV is more effective in several aspects, such as throughput, end-to-end delay, packet delivery ratio (PDR), energy consumption, jitter, packet loss ratio, and network overhead. Furthermore, this paper presents this systematic review based on AODV modifications in the Preferred Reporting Items for Systematic Reviews and Meta-Analyses (PRISMA). It also provides a methodological framework for the papers’ selection.

## Introduction

Wireless networks have attracted much interest from researchers and industries alike. Wireless networks typically contain several wireless nodes along with access points (Jabbar & Salman, 2021). They are multi-hop wireless networks built using static wireless mesh routers seamlessly linked through mesh-like construction. The decentralized nature allows every node in the wireless network to act as a transmitting node and be involved in data forwarding through the neighboring nodes. Mobile *ad-hoc* networks (MANETs) can be regarded as a kind of wireless mesh network (WMNs), specifically mesh networks. WMNs have become an important way to deal with the problems of next generation networks, such as giving service providers and businesses flexible, adaptable, and re-configurable architectures that are also cost-effective (Al Saeed, 2021; Kok, 2015; Razooqi & Al-Asfoor, 2022). All access points (AP) are attached to the wired network in the old version of the Wi-Fi network. But in wireless mesh networks, only one access point of the subset is attached, which needs the connectivity of a wired network (WN) (Wzorek, Berger & Doherty, 2021). When the access point connects with the wired network (WN), the situation is called an internet gateway (IGW). When access points (APs) are not connected to the wired network, the situation is known as mesh routers (MRS). The mesh routers use a lot of hop communication because they connect with internet gateways (Shi et al., 2019).

Current research in relation to WMNs concludes that the application of this network is used in community monitoring, surveillance systems, security, management, rescue operations, and also in-home networking (Muzammal, Murugesan & Jhanjhi, 2020). IEEE’s current wireless networking protocol technologies are described by 802.15, 802.11, 802.20, and 802.21 standards. All these are easy to apply to WMNs. The most common and widely used routing protocol is *ad-hoc* On-Demand Distance Vector (AODV), which is used in WMNs (Karri, Solanki & Khan, 2020). This protocol standardised the four additional protocols used by the IETF MANET working group. It creates new protocols based on the protocols of wireless mesh networking routers. Hybrid wireless mesh protocol (HWMP) is one of the four protocols that standardised WMN and uses the first version of IEEE (802.11s) (Höfner & McIver, 2013; Bello & Steiner, 2019; Shagdar, Nashashibi & Tohme, 2017; Feng et al., 2020).

IETF RFC3561 makes the details of the AODV protocol standardised (Garcia-Luna-Aceves & Hemmati, 2020). According to English composition, its details are uncertain and conflicting. The difference appears in implementing the AODV routing protocol (Van Glabbeek et al., 2013; IEEE Standard for Information Technology, 2007). The AODV routing protocol does not support the mobility of the reactive protocol (RP) and works with *ad-hoc* networking (Merro, 2009). The semantic procedure is also mixed with Dynamic Source Routing, Destination Sequenced, and Distance Vector protocols. It maintains and creates a route when it needs a single-path route.

The routing philosophy depends on two procedural syntaxes: route maintenance and route discovery (Hussien & Matloob, 2018). The AODV is established, and its process depends on the Handshake Communication Mechanism (HCM). Therefore, it’s used in broadcast, trio of uni-cast, and multi-cast to flood route requests (RREQ). Node has a single path to its destination (Perkins & Chakeres, 2013). The routing protocol of the AODV starts its work when RREQ (packet traversal broadcast route request) needs a valid link or path for the destination of neighbours searching (Pirzada, Portmann & Indulska, 2008). The source and destination nodes are attached to this routing protocol. The routing protocol replies to the uni-cast route (RREP) with the opposite unidirectional path of the source code. The valid path or link is maintained and saved between the source and destination nodes as required by the source node. The whole process is based on the source node. The source node’s life starts at the beginning of the request for route and node of destination. If the link is broken down during the process, then the route finishes all the process steps and creates a new communication link.

The communication link could break due to the mobility of the source node or a power outage. The analytical techniques of traditional AODV and other AODV-based protocols are a copy of the bed test practical (Merro, 2009; Hussien & Matloob, 2018; Perkins & Chakeres, 2013; Pirzada, Portmann & Indulska, 2008; Ramachandran et al., 2005). Specifically, the process of quantitative evaluation has some correctness qualities in its evaluation basic protocol. Experiment evaluation is a time and resource intensive process. The long evaluation process can consider only network scenarios of the defined set. It has no clear, beneficial, and correct behavior in the wide domain of deployment scenarios (Ramachandran et al., 2006). This issue is also found in the current routing protocol of the AODV. Its issues have been under analysis for many years (Sharma et al., 2020).

This research aims to increase the performance and reliability of networks and reduce the “time-to-market” of WMN protocols. The advantage of AODV compared to other routing protocols is that it needs fewer resources because the control messages and the routing tables of AODV are small. This makes AODV a better option for restricted bandwidth and computational resource systems.

Another goal of this work is to present a conceptual framework for MANET routing protocols based on various technologies and concepts. Several *ad-hoc* routing protocols have been suggested, such as AODV, Dynamic Source Routing (DSR), Optimized Link State Routing Protocol (OLSR), Destination-Sequenced Distance Vector (DSDV), *etc*. This article proved the claim that the AODV is an essential routing protocol. In addition, this work involves the recent advancements, review analysis, and extensions of the AODV and the concepts of modification to the AODV Routing Discovery Mechanics. Moreover, this work aims to give students and researchers a better outline of the present state of the art in this way. This work takes a systematic approach with the hope that it will help the researcher learn more about the problems that still need to be solved in the field.

## Literature review

This chapter started by describing studies that had been done before on the limitations of routing in mobile *ad-hoc* networks. The mobile and dynamic nature of WMNs could result in link breaking. This link breakage occurs when the speed of WMN mobility increases. Link breakage is especially common in reactive routing protocols like AODV. In the reactive protocol, the message of the broadcast would have found its way to the destination. When many nodes attempt to find a path along one route, the situation is called “route overloading.” The result of this overloading is the breakage of the link. When the link is broken down at the intermediate position of the path, then it finds a new local path that does not sort at the end-to-end route. Finding a new local path is advantageous for the message. Local repair process approaches have been covered by several scholars, including (Jinkang, 2020; Xiaokui, 2020; Gupta et al., 2020; Majumder & Roy, 2020; Lin et al., 2010).

Chekhar et al. (2021), presented a novel approach called WAODV (WAIT-AODV) which allows self-administration of nodes. Nodes are free to decide on re-transmitting RREQ, discarding it, or waiting before broadcasting it to their neighbours. Decisions are made based on neighbour statistics. This plan stops some nodes from broadcasting as much, which makes the network less crowded in the long run. stage of local recovery of its neighbours. The bypass and DSR are the same with extra delays and overhead. The stale route in DSR does not clarify the solution.

The combination of AODV and bypass file is used in a cross-layer of MAC-interaction (Alshanyour & Baroudi, 2008). To identify the loss of transformation of the packet, the routing layer’s local repair starts to work. The setting of the bypass is established when there is a failure link repair with the permission of the upstream node. This connection is established between the bypass and the downstream node through another node. The bypass minimises the radius of typologies and routing overheads.

The previous knowledge provides the schemes of route repair for the AODV process when links have failed. None of the available sources explain the comparative mechanisms of the Quality of Service (QoS) is crucial on any network. Agent-Based AODV (AB-AODV) manages trusted information locally with a minimum of extra messages and time delays (Bairwa & Joshi, 2020). Each node uses a multi-agent system with two agents—a monitoring agent and a routing agent. A new formula for trust value computation is proposed.

Kurian & Ramasamy (2021), they have proposed a trust-based secure path selection scheme. Trust values are reduced for flooding or non-cooperative nodes. Simulation results show that this scheme reduces the control message overhead by 4% and the service discovery ratio improves by 13%.

The authors have modified the traditional working mechanism of AODV as suggested in Islam & Shaikh (2008), and RREQ and RREP are now called SREQ and SREP, respectively. The proposed method utilizes two-hop service information. Many parameters, such as energy, mobility, and RREQ message broadcasting, limit the effectiveness of an *ad-hoc* network. AODV does not take into account the node energy in the route discovery process. Nodes with critically less energy can cause transmission failure and packet dropping. That ultimately re-initiates the RREQ and consumes more energy. To address this issue, an energy-aware protocol was proposed by Divya & Srinivasan (2021). The proposed scheme uses ant colony optimization (ACO) with a bacterial foraging algorithm (BFA).

Anantapur & Patil (2021), proposed an ACO based advanced secure routing scheme in MANET. The optimal route is determined based on residual energy, trust, degree of a node, and distance; these four parameters determine the fitness of each route using ACO. This modified AODV based on ant colony optimization (MAAODV-ACO) seeks to minimize the delay and improve the PDR. And the algorithm also aims to achieve a secure transmission path from source to destination against blackhole attacks.

The Hop-Power based AODV (PH-AODV) was presented by Ket & Hippargi (2016). In this approach, the selection of an active route depends on two parameters: energy and the hop count of neighbour nodes. The protocol was tested and compared with traditional AODV. The experimental outcomes show the superiority of HP-AODV in terms of end-to-end delay. Both protocols were compared in several different situations, such as networks with different sizes and speeds of mobility.

Dsouza & Manjaiah (2020), presented the reverse AODV (RAODV) protocol. It considers the active load of the path in the route discovery process and a less congested path is chosen. In RAODV, on receiving RREQ messages, the destination node broadcasts RREP messages instead of uni-cast in normal AODV protocol. The topology of MANET is very dynamic as nodes are joining and leaving the network on the fly. It is essential to broadcast the new route demand when a node joins the network. This broadcast increases the network congestion significantly if more nodes join and broadcast the messages.

Alani, Abdelhaq & Alsaqour (2020), presented a modified version of AODV called AODV-Velocity and Dynamic (AODV-VD). This novel scheme reduces network overhead and the route discovery control packages. Each node is classified as a reliable node (RN) or an unreliable node (URN). The class is determined by the nodes’ initial probability-based velocity vector. Reliable nodes have high broadcasting capabilities compared to unreliable nodes.

The Optimized Adaptive Multi-path AODV (OAM-AODV) proposed in Deepa, Krishna Priya & Sivakumar (2020), utilizes the alternate path to transmit the data on link break. The proposed method predicts the probability of link failure and auto switches to another optimal path and continues transmitting data. That ultimately results in a number of route discoveries and improves the throughput of the network. As there is no need for route discovery every time there is a link failure, it also reduces the packet drop ratio.

Energy-efficient protocols are important due to limited energy participants in a network, especially when Blue-tooth devices are part of the network. *ad-hoc* networks are infrastructure-less, self-administrated architectures. The administration of the network is highly controlled by the flood of messages. Communication between nodes cannot be done without energy consumption.

Ghori et al. (2021), presented the Optimized AODV (O-AODV) protocol, which beats BLE mesh and conventional AODV protocols on the scale of overhead, end-to-end delay, and average per-hope one-way delay. It also enjoys a 13% better packet delivery ratio compared to the AODV protocol, but it is only 9% less than that of the BLE (flooding) protocol. O-AODV also achieves better route requests and route reply times.

Network life time and route stability are directly affected by energy constraints and the mobility of the nodes in hybrid networks. These facts are typically not considered in conventional routing protocols. In Shi, Chai & Liu (2017), the authors presented the Regional Energy and Mobility Aware (REMA) routing protocol. The degree of dispersion is also considered to decide the route. An area having more remaining energy and a lower degree of dispersion is selected for network services. This helps in increasing the network life span. Another parameter to consider for a balanced network is the mobility of the node. Nodes moving with less average and having less movement dispersion across the network are considered stable nodes. Unstable nodes lead to high link breaks. However, the single radio client was considered for the experiment. The work can be extended to multiple clients.

Localization is a very important task for any Mobile Low-Duty Wireless Sensor Network (MLDC-WSN). Bethi & Moparthi (2022), presented gossip based on the demand distance vector protocol. This improved version of the protocol eliminates the redundant information for the next node discovery. The gossip AODV handles the issues created by the clock drift on nodes. The experimental results show the improvement in multiple parameters, including discovery delay, wake up time, and energy consumption.

Neighborhood density AODV (ND-AODV) helps in reducing the overhead of ever-changing WANETs, which are highly dynamic and scalable in nature. An expected transmission time (ETX) is used to improve the reliability of the conventional AODV protocol instead of simply a hop count. The RREQ is greatly reduced by considering the neighbours’ density. The derivation of optimal density is empirically shown in the literature. The proposed method is compared with AODV and probabilistic AODV on various performance metrics such as overhead, PDR, throughput, jitter, *etc* (Malnar & Jevtic, 2022).

The Internet of Vehicles (IoV) is getting more attention day by day with the technological advancement in the Internet of Things (IoT). VANET makes communication possible between moving objects in an infrastructure-less environment. Drones and other unmanned aerial vehicles (UAVs) are useful for a variety of applications such as surveillance, shooting, security, and monitoring. The authors have presented drone assisted DSDV (DA-DSDV), drone assisted OLSR (DA-OLSR), and drone assisted AODV (DA-AODV). Experiments were conducted on grids of sizes of 300 × 1,500 m and 300 × 6,000 m. The drone assisted protocols have achieved quite good results over conventional algorithms (Afzal et al., 2021).

The Internet of Underwater Things (IoUT) is gaining popularity due to factors like exploring the ocean surface, security, industry expansion, military *etc*. Bhattacharjya, Alam & De (2019), presented an energy-efficient architecture for the Underwater Wireless Sensor Network (UWSN). A prolonged life span is very crucial for wireless networks. The work focuses on the design of energy-aware systems that require minimum energy to operate. It uses the concept of multi-hop transmission. Work also evaluates the performance of various protocols such as AODV, Zone Routing Protocol (ZRP), and Interzone Routing Protocol (IERP). Algorithms are analyzed on different packet sizes for metrics such as average jitter, throughput, packet loss, energy requirements, average delay, *etc*.

A decision factor (DF) based modified AODV (Mo-AODV) is proposed in Pandey & Singh (2021). The estimated deciding factor is the residual energy of the node and the signal power. Hello, messages are sent periodically to get the energy status of neighbour nodes. On receiving a hello message, the node updates its table if the residual energy of the sender is above the predefined threshold. This mechanism avoids unnecessary broadcasting of route requests. MANET is very highly dynamic in nature in terms of topology, mobility, scaling *etc*. It is also constrained by a lot of other things, including the energy of the node, latency, and reliability. At any given moment, the node would be in one of the following states: transmit, receive, idle, or sleep. The node consumes the most energy while transmitting data. All nodes are battery operated and have limited energy backup.

Energy-efficient protocols are always a need for MANET. Enhanced AODV (ENH-AODV) focuses on route selection by taking into account the quality of links and upcoming nodes (Pandey & Singh, 2022). Each node maintains a list of efficient neighbor nodes based on their energy level. Wireless sensor networks operate in the industrial, scientific, and medical (ISM) band, which is prone to interference, frequency overlapping, coexistence, *etc*., which leads to performance degradation.

Joon & Tomar (2022), suggested the Energy Efficient Q-learning AODV (EAQ-AODV) protocol for efficient routing. Cluster head selection is done through the reward mechanism based on Q-learning. Q-learning based AODV enables the optimal path selection considering various factors such as residual energy, communication range, licensed channel, number of hops, and trust factor. The work can be extended to mobile nodes and the interference introduced due to the mobility of nodes can be explored further to improve the work.

To address the issues of congestion and energy, Wu, Wei & Li (2021), presented the Multi-Objective Simulated Annealing AODV (MOSA-AODV) routing mechanism. These two metrics are used to select routers. The proposed method’s results are compared to existing approaches RA-AODV and IEEAODV. The MOSA-AODV protocol has a good result in extending the network’s life span. However, the MOSA-AODV model still has a network overhead.

Path congestion and path robustness were used to evaluate the fitness of each solution. Path congestion reflects the load of the route, and path robustness reflects the life span of the route. The survival time of a path is defined by the minimum energy level of each node in the route.

Improved Priority Aware AODV (IPA-AODV) targets the issue of the velocity of mobile nodes in the network. The wireless network does not allow mobility beyond 2 m/s. The proposed method restricts any node from participating in the routing if its velocity is greater than the predefined velocity threshold (Perumal, Prabhu & Selvi, 2022).

In Choudhary et al. (2022), the author presented a fuzzy logic-based AODV that considers various parameters such as residual energy of the node, the expiration time of the link, speed, hop count, and bandwidth to determine the optimal path. The trust value computed this way is used to select the next node. The multi-metric based fuzzy approach reduces the probability of link failure. The fuzzy rule base is designed based on the stated five parameters to estimate the value of trust of a given node. All five inputs are encoded using binary linguistic variables, and the output (trust of a node) is encoded in five different linguistic values. Seven fuzzy rules have been devised to compute the trust value. More metrics and more factors can be considered to enhance the work in the future.

Quality of Service (QoS) has been the focus of recent attention by Avudaiammal, Vathsan & Sivashanmugam (2022). A QoS-driven *ad-hoc* on-demand distance vector (QAODV) driven routing protocol has been proposed. A power constraint factor is used with the distance parameter. Experiments have shown an improvement of 14.28% in throughput, 27.83% in delay, and 11.6% in packet loss compared to conventional AODV. RREQ packets are broadcast throughout the network, and power and hop count are updated at each node. On receiving back the RREP, the destination node checks the power of the node. The routes that require the least amount of energy are eliminated. The route is selected for further communication if its power is greater than a predefined threshold. If the power of a neighboring node goes below a threshold, route discovery is initiated again. Work can be extended further for mobile nodes. AODV protocol’s real-world implementation in RFC (Perkins, 2004).

Traffic load effects, source-destination pairs, and mobility in different stages have been discussed. Reports in relation to routing protocol reviews and surveys can be found in the literature. There are many reports on popular routing protocols; this protocol depends on different routing mechanisms (Saini & Sharma, 2019; Walikar & Biradar, 2017; Alotaibi & Mukherjee, 2012). Some reports cover only the protocols that depend on parameters (*e.g*., energy, delay, and bandwidth). Many reports are incomplete and have the minimum details of protocols because many protocols are present in Jhaveri & Patel (2015). Some specific protocols are described that contain important factors relating to protocols. The current study is based on the report of AODV’s work.

The survey study is based on the AODV and its analysis. In short, it has been defined that many survey reports cover famous extensions and the formation of the AODV protocol. This search report gives a quick overview of how the AODV routing protocol is used in WMNs and in other research. Agents are the tiny packets in-network, which move across the network and collect the data bout networks and the node.

This data is used to improve various metrics of the network. Agents have characteristics such as autonomy, mobility, intelligence, compatibility, collaboration, *etc*. These characteristics allow the agent to freely move, communicate, and gather information. Authors in literature presented an improved version of AODV, which is known as Agent-based AODV (A-AODV), which operates on three principles: routing metric, route discovery, and routing algorithm. Simulation results show that A-AODV always has better PDR compared to AODV and DSR (Nguyen et al., 2021).

Several limitations still exist with the new versions of AODV, such as broadcasting issues, insufficient path discovery, data packet duplication, longer routes, and no control of information distribution. Also, the network delay and overhead in the related work were noticed due to the discovery routing operation.

## Conceptual design

The current section of the systematic review begins with the layout of the wireless mesh network, including the topology and routing mechanisms. Furthermore, the conceptual framework for the AODV routing protocol and the modifications to the AODV routing protocol will be discussed in this section.

### Routing protocols

**Figure 1:**
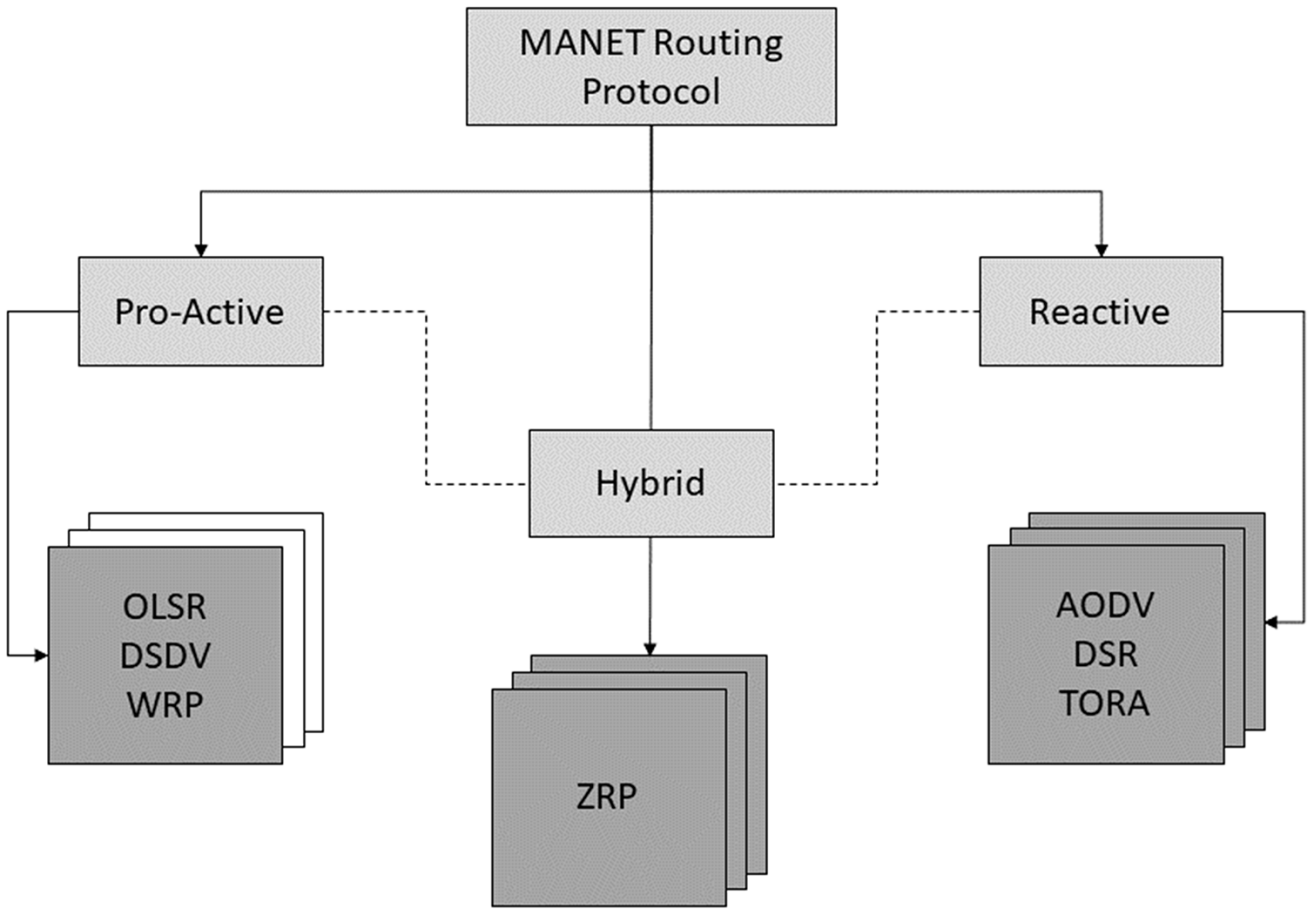
Routing protocols classification.

Routing protocols can be either proactive, reactive, or hybrid (Cucor, 2021). As the name suggests, reactive protocols only discover the path when needed. Dynamic Source Routing (DSR) protocol, *ad-hoc* On-Demand Vector Routing (AODV) protocol, *etc* (Kakamoukas, Sarigiannidis & Economides, 2022). This type is known as “on-demand or reactive routing protocols.” In proactive routing protocols, nodes keep updating themselves about the route by periodically sending messages to neighbour nodes. Examples of proactive routing protocols include the Optimized Link State Routing (OLSR) protocol, the Destination Sequenced Distance Vector (DSDV) protocol, and others (Grover, 2022; Rovira-Sugranes et al., 2022). Routing protocols based on the nature of their operations are depicted in Fig. 1.

For DSDV (Li, Wang & Yang, 2010), messages are exchanged between mobile nodes present within the range. Routine and triggered are updated as a result of routing. Updating is started when the information is exchanged between the routing table and the neighbour force. If the node releases a packet but the destination is unknown, the routing queries are sent out to that packet present in the route. The data packet stores it temporarily until the destination node receives the reply. When a package is sent, the buffer maintains the size and time of that packet according to its system. The packets that know the destination route without any routing query help are routed directly. The destination is not found in some cases, so packets reach the default destination, called a “routing agent.” The routing agent creates the next hop for the packet.

The dynamic source route protocol controls the source route of data packet information *via* the agent node (Torshiz, Amintoosi & Movaghar, 2008). Each router receives the data packets. It sends to the source route if no information from the routing packet is present for the packet’s destination route. It also sends route queries if the packet is unknown; it is the destination. At the initial stage, the routing query is delivered with the help of nearby nodes. Significantly, the question is sent to those packets that do not have information about the destination route. Suppose information about the destination route is found, and then the reply route moves back. The DSDV and DSR protocols are combined with the AODV protocol (Zhang & Gulliver, 2005). AODV works on the DSR maintenance- recovery route. It also takes the properties of the DSDV protocol like beacons and routing sequences hop-by-hop.

An RREQ is created when a node needs the destination route. The middle node sends RREQ and also makes a reserve route of destination. The request for the destination route reaches the node, which then creates an route reply (RREP). This contains the hops that are needed to reach the destination route. All nodes participate in sending the reply, and then the source node makes it to the destination of the forward route. This creation is not meant to create a complete route compared to the source route but to form the hop-by-hop state destination of the source route. The Optimised Link State Routing Protocol (OLSR) knows all the present routes. OLSR is an advanced type of “pure link-state protocol.” The topology changes, and then the OLSR overflows to the topological information of an active node. An Multi-point Relay (MPR) is used to reduce the overhead of network protocols (Hasan et al., 2018).

The MBR decreases the copy re-transmission of the forward broadcast packet. In this process, re-transmission bounces to the small neighbouring nodes (Darabkh, Alfawares & Althunibat, 2019). Instead of using all its neighbours’ nodes, it chooses the small set covering the network region (radio range of one-hop). There are two types of OLSP control messages: The message “HELLO” determines information about hosting neighbours and links. The topology control message sends the neighbour’s information containing the MPR selector (Darabkh, Alfawares & Althunibat, 2019).

### *Ad-hoc* on-demand distance vector (AODV) routing protocol – overview

The routing protocol decides how the routers communicate to deliver information that allows them to choose routes among any two nodes present in a computer network. The AODV routing protocol avoids the routing loop issue in *ad-hoc* networks (Yan & Chung, 2020; Abbas et al., 2020). Because of their self-starting behavior, they do not require any stimulation and operate in an environment with several mobile nodes capable of withstanding various networking etiquette such as packet loss, link failure, and nodal mobility (Taterh, Meena & Paliwal, 2020).

The routing protocol regularly maintains a routing table at every node. The routing table contains the hop count, sequence number, and the next hop node. The hop count specifies the present distance to the destination node. All the data packets approaching the destination node will be forwarded to the next hop node. The sequence number reflects how fresh the route is and contains a time stamp (Goyal, Sharma & Kumar, 2020; Goyal, Goyal & Kumar, 2020).

The AODV protocol lets the mobile nodes in the network acquire the routes swiftly to fetch new destinations and eliminates the route maintenance of nodes to destinations that are not currently active. It enables the mobile nodes to acknowledge periodically if any change is detected in the network’s topology or if any link breakage occurs (Chaturvedi & Kumar, 2021; Marcano, Nørby & Jacobsen, 2020).

The AODV protocol also informs the affected nodes during link breakage so that they can refute the routes through the lost link. The AODV protocol authorises the destination to create the destination sequence number, which is added with any of the route information sent by the destination to its requesting nodes (Kamboj, Dalip & Rai, 2020).

### AODV routing methodology

**Figure 2:**
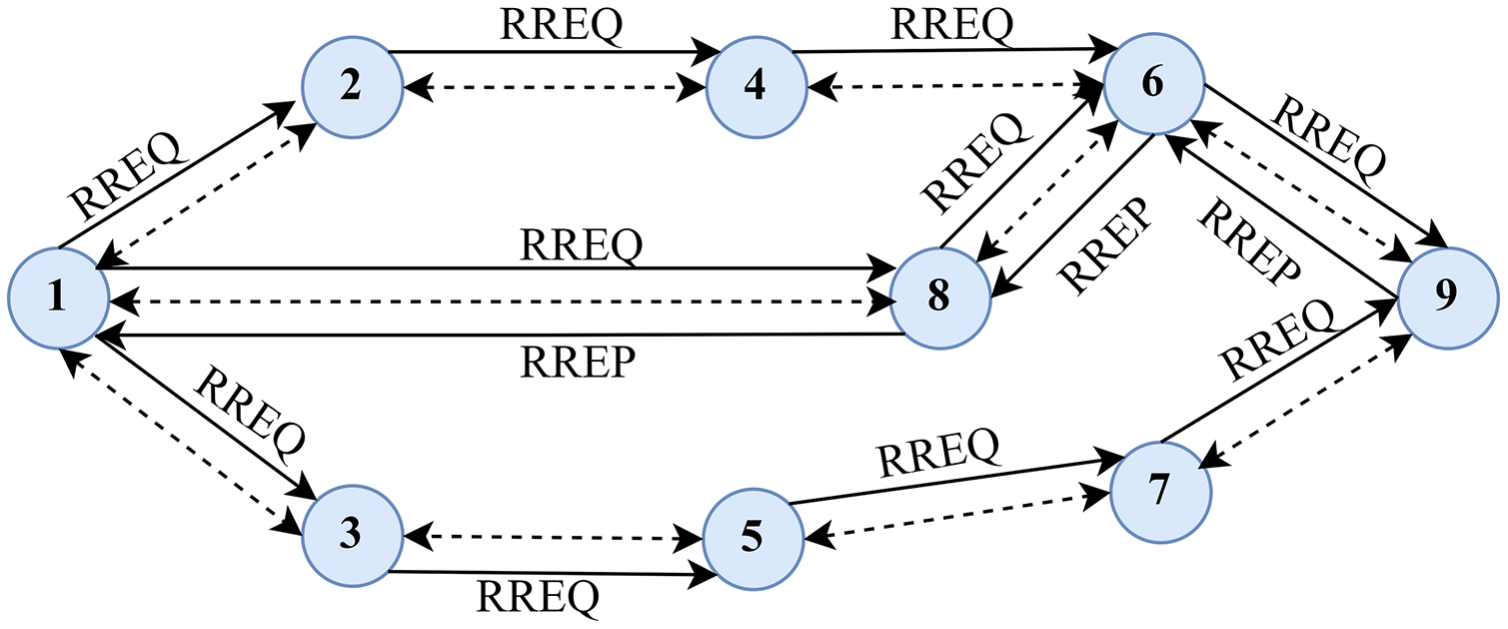
Typical routing methodology in AODV.

AODV follows a hop-by-hop routing methodology. Figure 2, shows the typical routing methodology followed by the AODV protocol. The source node initiates the route request, or RREQ, to find the route to some destination. Upon receiving the request, the intermediary nodes transmit the RREQ and establish a contrarian route to the specified destination (Hanan, 2020). The node that contains the destination route acknowledges it with a route reply (RREP) after receiving the route request. The RREP contains a sufficient number of hops to reach the destination. The nodes are responsible for acknowledging with an RREP that the source node also establishes a forwarding route to the destination (Soomro et al., 2020; Sayedahmed, Fahmy & Hefny, 2020). Thus, AODV establishes a route between a source and a destination through a hop-by-hop routing methodology.

The overall process of AODV routing mechanism can be divided into two main stages:

#### Discovery of routes

During route discovery in WMNs, the source node will transmit a packet to a particular destination node to verify with the routing table whether it contains the present route to the node because the legal route message from source to destination will be present in its routing table. If route information is present in the routing table for that node, it transmits a packet to the appropriate next hop node towards the specified destination. If the node does not contain the route to the selected destination, the source node initiates route discovery to begin a presentation towards the selected destination node (Perkins, 2008).

To accomplish the route discovery process, the node generates an RREQ packet containing the IP address and present sequence number of the source node, the IP address, and the previous familiar sequence number of the destination node. The RREQ packet will also contain a broadcasting ID, which gets incremented whenever the source node creates an RREQ packet. Once the node generates an RREQ packet, the source node forwards the RREQ packet and starts a timer to wait for the acknowledgement.

**Figure 3:**
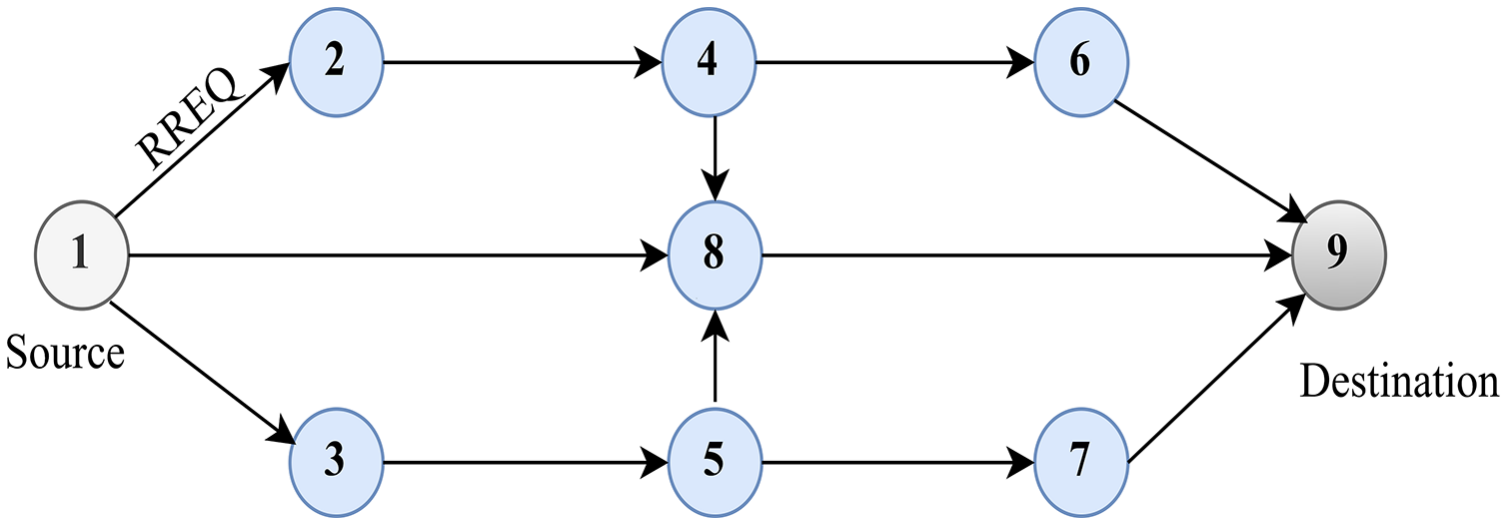
Route discovery in AODV.

Figure 3, shows the route discovery process in AODV. Once receiving an RREQ packet, the node initially verifies the identity by checking the source node’s IP address and broadcasting the identity pair. For every received RREQ packet, each node has to maintain the log of the broadcasting ID and the IP address of the source node for a finite period. To acknowledge the RREQ packet, the node, through its routing table, should be familiar with the route to the destination. The RREQ will also notify the sequence number of the destination, which avoids the loop formation in routing. This assures the former intermediary node that the returned route is not old. If the node fulfils the requisites mentioned earlier, it acknowledges by uni-casting the RREP packet to the source node.

#### Maintenance of routes

After route discovery between a source and a particular destination, node mobility in the wireless network will influence only the routes that contain those nodes on an active path (Perkins, 2008). If the node movement of the source node is found during data forwarding, it has the option of restarting the route through discovery and constructing a new route to the destination. A RERR or route error message will be sent to the affected source nodes. Initiated by the node upstream, it recites the destinations which are not reachable currently due to the list link. A RERR message will be sent to the neighbouring nodes. Upon receiving the RERR message, the neighbouring nodes note their path as an inoperative one by making the destination node value infinite. If the source node receives the RERR message, it restarts the route discovery process to find the routes while in need.

**Figure 4:**
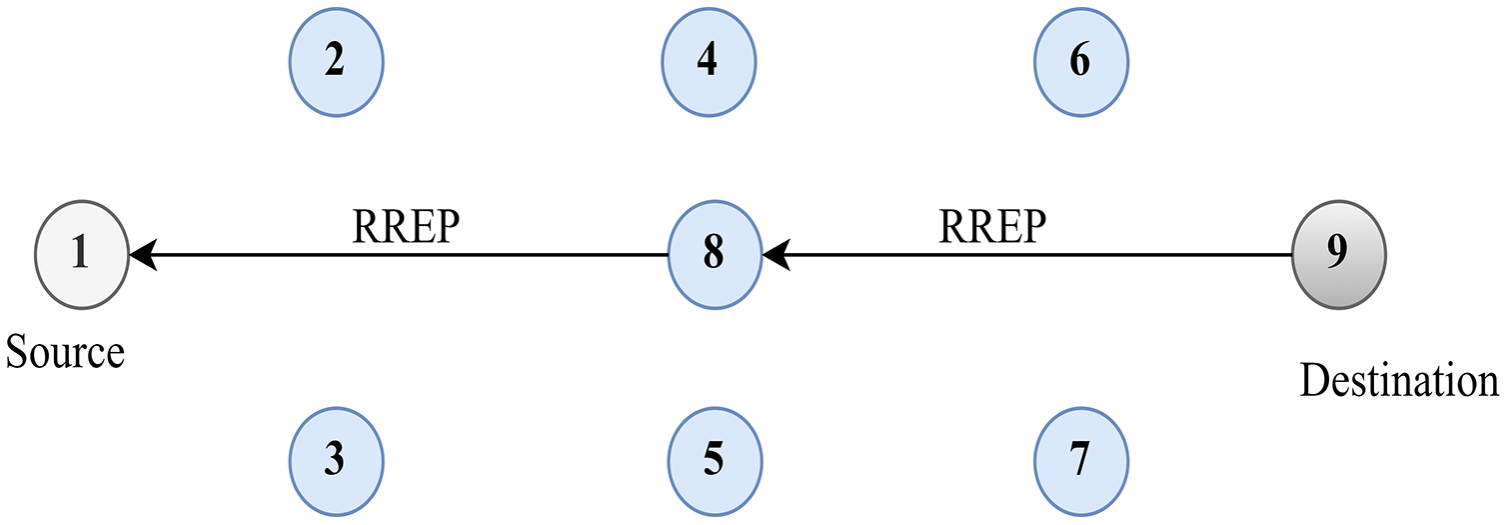
Route discovery.

Figure 4 shows the source route discovery process. Route maintenance can be done using two methods. In the first method, the source node forwards the RREQ message to its neighbouring RREQ with the IP address of the source and destination along with the sequence number and broadcasting ID. If the destination node sends the RREP message, the source node receives the message through an intermediary node.

However, another method of route maintenance can be done locally through an intermediary node that recovers the broken link. The intermediary node forwards an RREQ message through its neighbouring node to the destination. Upon receiving the RREQ, the destination acknowledges the intermediary node with an RREP message that again establishes the route between the source and the destination.

### Wireless mesh networking

**Figure 5:**
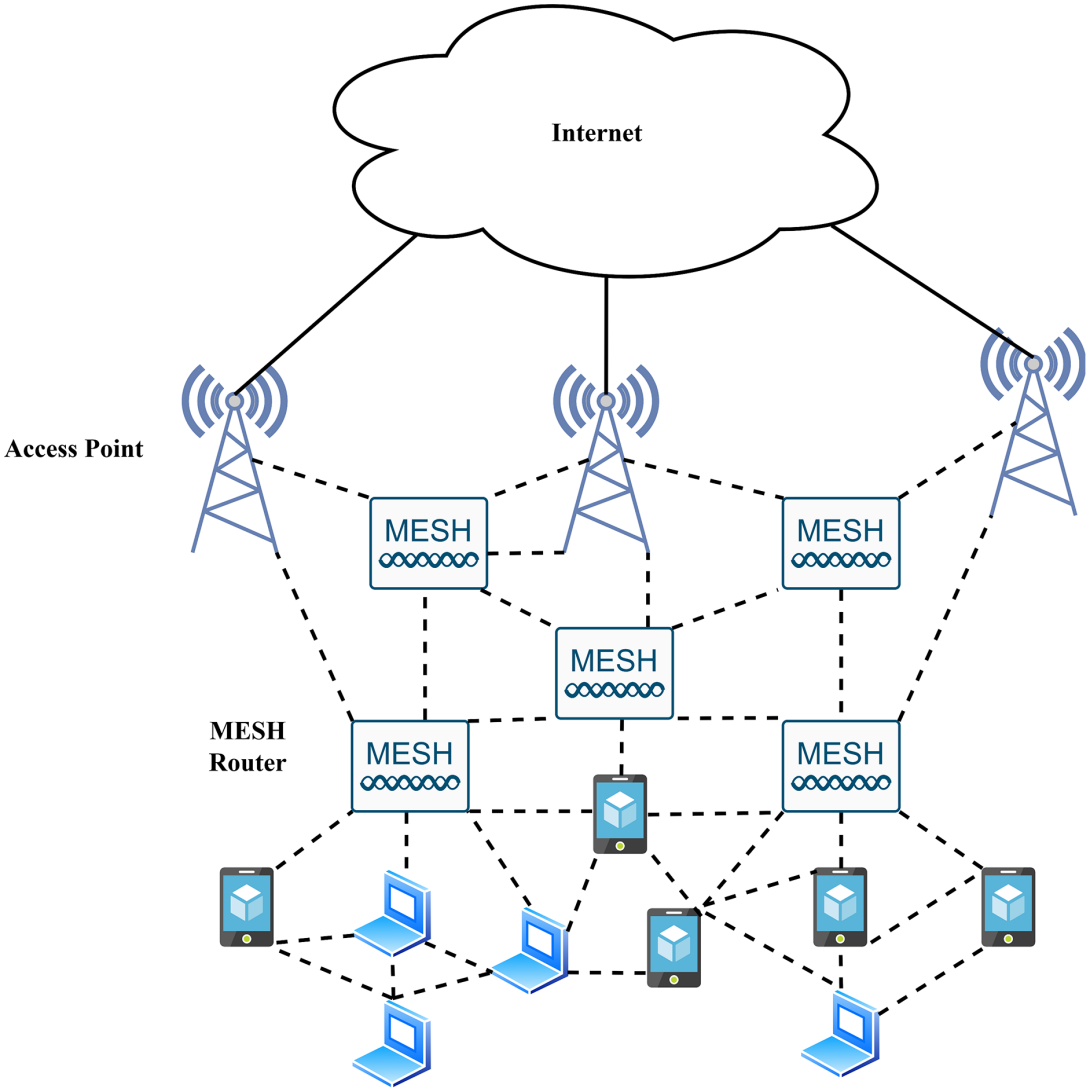
Wireless mesh network.

As shown in Fig. 5, the radio nodes in WMNs are often categorized into groups of mesh clients and mesh routers (Kaur & Kaur, 2020). Each node exhibits both host and router functionalities. The network range is extended to the nodes that lack a direct wireless transmission path with multi-hop communication techniques (Kaur & Kaur, 2020). A wireless mesh router serves additional routing functions compared to the essential gateway/repeater functions of a conventional wireless router. Furthermore, many mesh routers possess multiple wireless interfaces (Roy & Debarshi, 2020). Mesh clients also contribute to the network as routers, but they do not have gateway or bridge functions and usually have only one wireless interface. The practicality of WMNs lies in the fact that they are self-organised and self-configured (Yagci, 2011).

A design aim for WMNs is to enable maximum mobility for the mesh clients, while the mesh routers are less mobile or static. These WMNs work extremely fast in every type of technology. This technology’s weaknesses include self-configuration, broadband capacity, self-healing, and self-organisation. Therefore, it would be beneficial to implement modern wireless *ad-hoc* networks (Cilfone et al., 2019).

WMNs are helpful because they make it easy to maintain and deploy reliable and flexible services at a lower cost than the alternative high-modular solutions. All these properties stress international agencies to create a high-level specification of IEEE protocol versions that are WMN of 802.11s, WLAN 802.11, WPAN 802.15, WMAN 802.16, and MBWA 802.20 (Britton & Coyle, 2011; Yagci, 2011).

### The topology and routing mechanisms of the WMN

**Figure 6:**
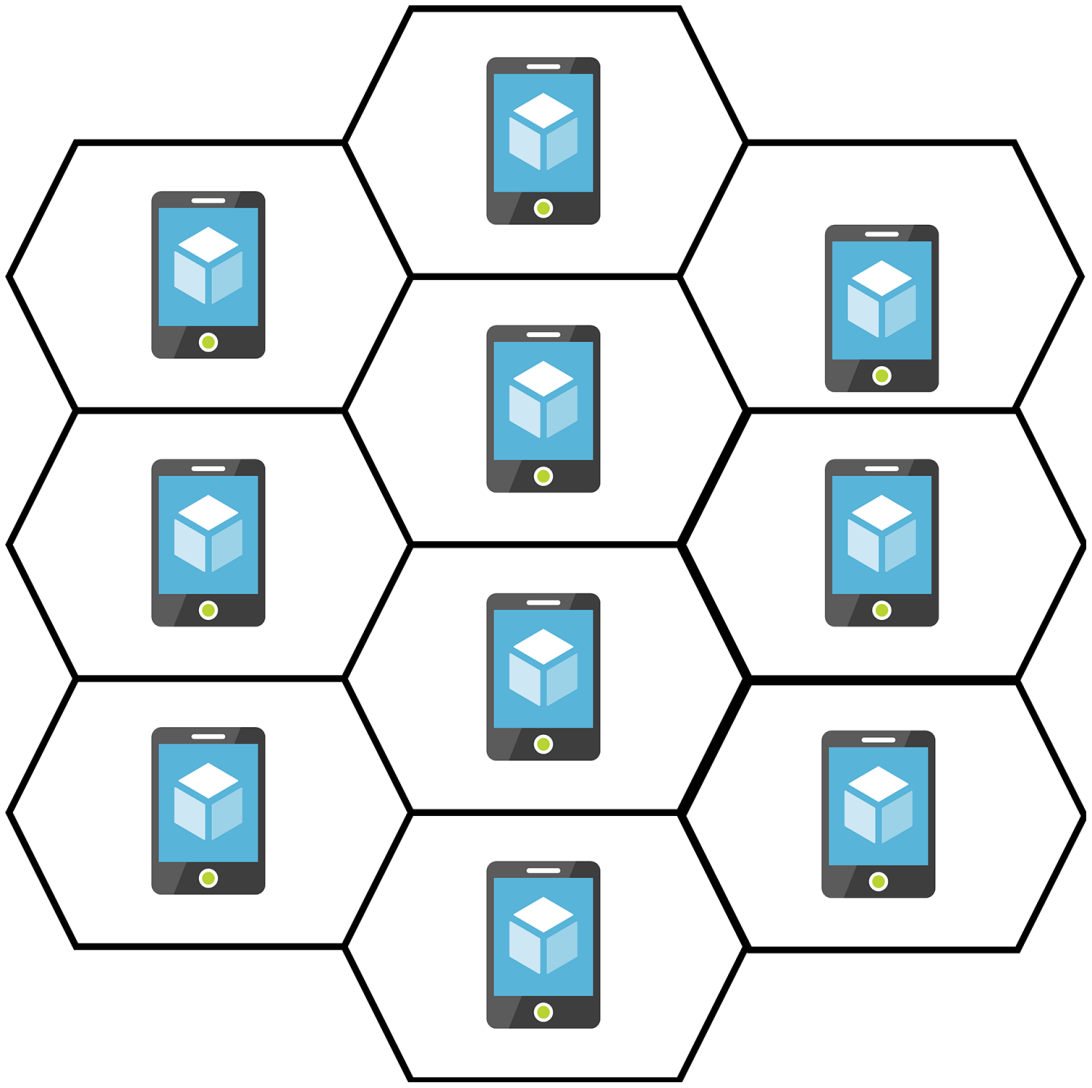
WMN topology.

So far, a comprehensive range of routing mechanisms has been discussed. This section narrows down the alternatives by interpreting the network topology. Figure 6, depicts the topology of our concern (Abolhasan, Hagelstein & Wang, 2009). This topology reflects a client W.M.N., where every node has precisely six neighbours, each of which is at the same distance. The hexagonal borders denote the neighbourhoods of the nodes. The nodes are stationary. Hexagonal geometry enables accurate measurements in the simulations, such as hop-to-hop delay, because of the equal distance between nodes. The selection of protocols to be tested in this topology and the reasoning behind their selection are described in the following sections (Abolhasan, Hagelstein & Wang, 2009). AODV’s unique feature for reactive routing protocols is its use of a table-driven routing framework. Reactive routing protocols such as AODV differ from proactive protocols like OLSR in performance (Perkins et al., 2001).

### Wireless mesh network routing discovery mechanics

Wireless networks are always prone to various attenuation sources. There are certain design principles to achieve an optimal routing mechanism for the WMNs (Mahfoudh & Minet, 2007). The most crucial focus is to be aware of network topology. Although the topology of a WMN continuously changes because the control scheme and topology discovery have shown fast performance. The alternative principle involves employing link metrics with a low hop count. A low hop count is a desirable metric because of its simplicity. It wants to generate interference and congestion between WMN nodes. The process of routing in the WMNs is grouped into two categories in Kushwaha, Gupta & Ghrera (2015), described below.

- **Routing protocol Predictive:** This routing method makes predictions about the traffic demand based on the previous network tendencies of traffic. It also gives a specific result dependent on this forecast.
- **Routing protocol Oblivious:** Network traffic improves with oblivious routing. This routing contains all accounts of potential traffic and considers the maximum case of traffic load.
- **Cross-layer:** Cross-layer design exchanges data between layers to maximize network effectiveness and adaptability. Knobs define each layer in a cross-layer design. Higher layers use these parameters to fine-tune their control settings based on the network’s current condition. The cross-layer structure design is typically posed as an optimization problem with variables and constraints from multiple layers. The layer control knobs’ values must be optimized.

Two important factors make the design of a cross-layer an exciting topic for WMNs (Rahman, O’Hara & Kruys, 2010). The first factor is to know the radio path and best node channel available in wireless communication. The second factor is mobility, which changes the channels of nodes that also change the radio paths. The switching process of the design cross-layer is fast between the channels.

### Modifications to the AODV routing protocol have been previously proposed

The nature of the AODV routing protocol is reactive. The core is based on the maintenance and recovery of the routing process. The process of discovery routing is started when the node sends a packet. It creates broadcast and RREQ packets when a node wants to release a routing packet. For that reason, modifications to the AODV protocol that concern the route discovery procedure are needed.

- **Probabilistic AODV (PAODV) protocol:** To solve network blockage generated by the protocol of AODV, a modification called the PAODV protocol has been developed (Tingrui et al., 2009). This protocol relies on the probabilistic technique. It determines whether the node should release the RREQ packet to the network or not. This protocol starts its work when it increases traffic in the network. Each node has a large number of adjacent nodes. Then the probability of protocol search multi-routes will be higher. In that situation, there is a decrease in the number of packets of RREQ sent to the network. This procedure is repeated several times until the network is less congested. The procedure also creates a high-level delay in the starting position. The primary factor is the RREQ packets sent with maximum probability at this point in the scant network. When the probability is low, the network is congested. The PAODV protocol’s overhead is minimal, especially when the network is overloaded. The network’s overload problem occurs when the protocol of AODV routing works partially (Bugarcic, Malnar & Jevtic, 2019).
- **Modification of AODV RREQ mechanism:** The standard process of the AODV routing protocol sends the route broadcast request to the total nodes. In the scheme proposed (Nissar, Naja & Jamali, 2015), a table node maintains the hop node. The subset of neighborhood nodes permits transmission of a message, and the selection of the node dynamically depends on the service properties and the application. Every node of a network sends a request route message when the neighborhood’s density condition is well placed.

The scheme proposed (Pumpichet, Jin & Pissinou, 2013), decreases the blockage of unnecessary network transmissions. The middle node receives the RREQ packet, which contains the path list in which this node’s path is a highlight. If selected nodes permit the sending of RREQ packets, then the path of these nodes is mentioned in the path list. By the proposed scheme, the middle node density is considered a decision to forward REEQ in specific criteria at the intermediate node. Based on this discussion, when the neighbor nodes increase, the node’s probability of transmission will decrease. Hence, it reduces the overhead of transmission. Unplanned selection of nodes from neighbor sets increases the chance of full network coverage. This achievement is only gained at the higher cost of complexity (Pumpichet, Jin & Pissinou, 2013).

c. **Neighbour PAODV (Nb-PAODV) protocol:** Some new features have been added to PAODV that solve the problem of the PAODV protocol called the Neighbour-PAODV protocol. The new features in PAODV relate to the addition of a parameter. This parameter shows the probability of rebroadcasting of the RREQ routing packet in the discovery routing process. It is evaluated by the following formula, as described in the study (Bugarcic, Malnar & Jevtic, 2019):

(1) $$p = 1/Nb$$

In the formula, Nb is the neighbor’s number of the node that sends the packet. In the AODV protocol, each node has a list of neighbors in its routing table. Therefore, it is easy to determine the “p” parameter during the execution. This type of probability solves the problem of the PAODV protocol. The probability of a rebroadcast RREQ packet in the network is based on the neighbor’s number. The probability is lower than the number of neighbors will increase or *vice versa*. It is not sectional to find out the mean calculation of neighbors in the network. Every node owns the probability (p) and the number of neighbors (Nb). The p of the node relates to the Nb of that node. The routing table has Nb numbers (Bugarcic, Malnar & Jevtic, 2019).

### The simulation of AODV routing protocol

To determine the order protocol’s performance, the search work has previously presented the benefits of the network’s simulation software. Simulation is widely used in the computer network field. The literature describes the network’s commercial and free reproduction tools and supports different levels for different purposes, like analysis and design of computer networks.

This search paper is not completed, but this section references initial cases to the readers. The open-source simulators category describes the report of Network Simulator-2 (NS-2) and Network Simulator-3 (NS-3), which are actual examples of literature. The Network Simulator-2 (http://www.isi.edu/nsnam/ns/) is the older version of the Network Simulator-3. Network Simulator-2 is widely used because it has some new and different tools. The implementation of NS-2 depends on discrete-event simulation.

A study conducted by Li & Peng (2020) evaluated the protocol ad’s performance and concluded that the algorithm protocol simulates NS-2. It is essential that the ant colony algorithm (AOC), AODV discovery algorithm, and DSR protocol are balanced. According to Li & Peng (2020), the NS-2 software simulates the current mainstream network. At the initial stage of the simulation group, the wireless nodes are scattered in a 1,000 m × 1,000 m rectangular region. Each node’s range of transmission is two hundred and fifty meters. The moving model is also known as the random waypoint model.

In simulation experiments, the source and destination nodes are generated in an unplanned manner. The lifeline of these nodes is set to 40s. The IEEE 802.11 communication protocol uses the layer of MAC. The average result of the simulation was 60 independent simulation experiments. This increase in the Fortified Ant Protocol is much better than the classic Ant Colony Procedure. The standard protocol of end-to-end delay and the transmission success rate are best from two protocols, AOMDV and AODV (Modi & Panchal, 2014).

In another study, Rajitha Tennekoon (Wijekoon et al., 2014), have proposed that the protocol be applied to the well-known simulator. Network Simulator-3 (NS-3) is the successor version of Network Simulator-2. NS-3 is the standard model of the real world. It is a simulator that provides the fundamental architecture of the environment. The more realistic simulator of NS-3 is not found in the present age. Tarapiah, Aziz & Atalla (2017), has discussed the different instruments to measure protocols’ performance. The implementation of routing protocols is an analysis of two mobility models. This model used NS-2 simulation instruments of the VANET network.

In the study of Kanakaris, Ndzi & Ovaliadis (2011), Network Simulator-2 is used to determine protocols’ performance progress. Simulations determine the routing protocol’s performance according to the size of networks like 10, 20, 30, 40, and 50. The speed of mobile lies between 1 to 40 m/s. Constant Bit-rate (CBR) transmission is used by all nodes over User Datagram Protocol (UDP).

In Osanaiye, Alfa & Hancke (2018), has discussed that Network Simulator-2 is a separate network from the simulator used in the implementation of performance compared with the MANET protocol. The first and basic implementation of AODV and NS-2 of the MANET process is to execute and find performance parameters.

The simulation network of the VANET network simulator 3.29 is used in the study of Bugarcic, Jevtic & Malnar (2019). NS-3 has a mobility model that uses the trace file of NS-2. At the initial stage increase, the nodes increase the protocol’s overall performance through the AODV protocols. Based on this, it is considered the best network for connectivity.

**Figure 7:**
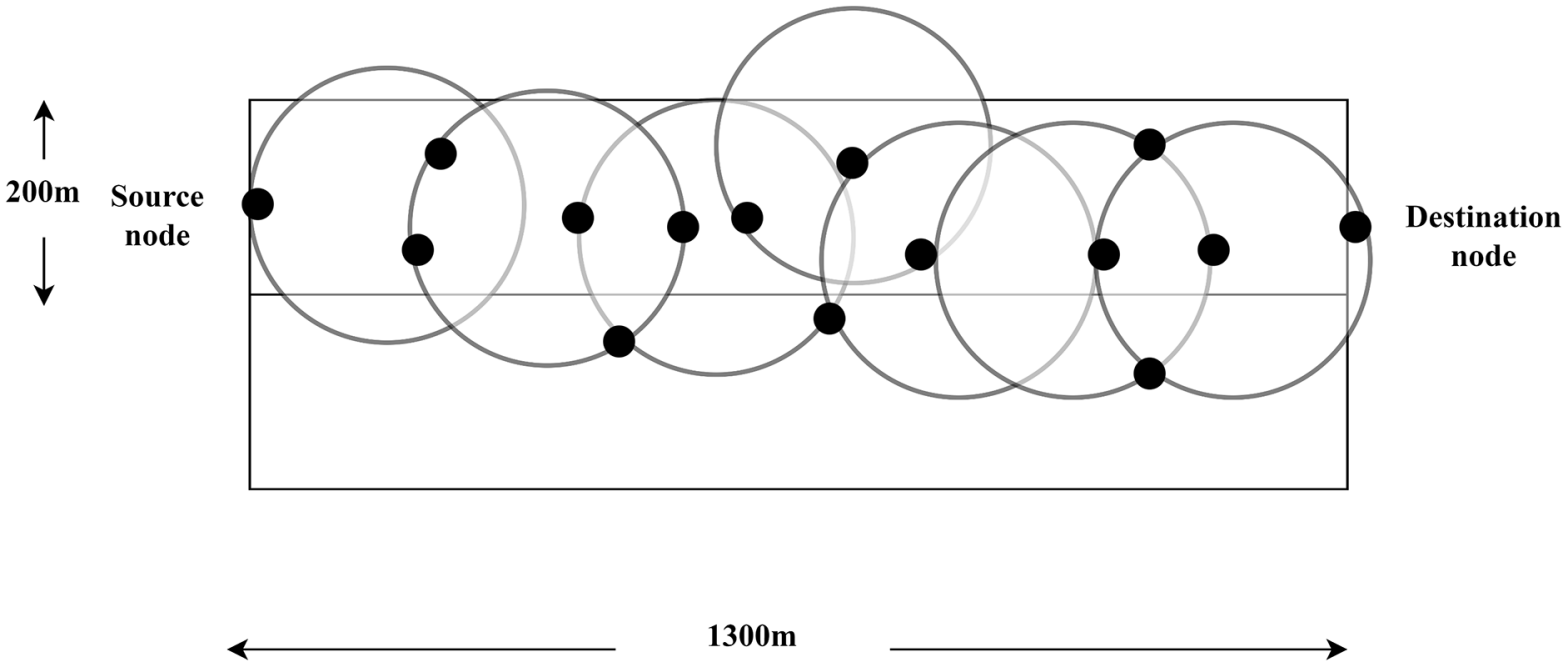
Source and destination node location.

Suhaimi, Mamat & Azzuhri (2010) has performed a simulation in his study using the NS-2, the version of 2.33. The total area of the simulation environment is 1,500 m × 300 m. In this area, fifty mobile nodes move randomly. As is represented in Fig. 7, the static source and destination are situated on both ends of the site (Suhaimi, Mamat & Azzuhri, 2010).

Recently, there has been an increasing amount of investigation into NS-2 by the authors. They proved by their work that the NS-2 is more flexible in scenarios and analyses of the network parameters. NS-2 outcomes are based on various network factors such as throughput, packet loss, packet delivery ratio, delay, error, and intrusion. Also, they described it as a good tool for experimenting with dynamic network typologies (Alameri & Komarkova, 2020; Kamel, Alameri & Onaizah, 2017; Alameri, 2019; Alameri & Komarkova, 2019).

Moreover, recent evidence (Issariyakul & Hossain, 2009; Alameri & Komarkova, 2022; Alameri, Hubálovskỳ & Komarkova, 2021; Alameri, Komarkova & Ramadhan, 2021), proved that the main advantages of NS-2 are that it does not demand expensive equipment, it can evaluate even the most complicated scenarios, outcomes can be achieved fast, and it allows for testing more concepts in a shorter period of time.

## Survey methodology

The presented work searched through the most significant search engines for scientific literature to comprehensively understand the subject. The submitted paper used Scopus as a primary source of information and combined the results of that search with the search results from the other databases. The decision to make this selection was based on the desire to take into account all major journals, conferences, and workshops that are either acknowledged by the scientific community as relevant to the subject at hand or are declared as appropriate by publishers or editors but are nevertheless screened and included by reputable indexes. Combining the results of the database queries yields the examined papers. This decision gives the suggested work more stability and guarantees the quality of the chosen articles because the authors did not include any informal literature, like informal reports, ongoing development or work, technical notes, or PowerPoint slides, which are notoriously hard to judge in terms of quality, as shown below.

### Protocol and registration

A systematic review took place along the lines of the Cochrane process. The research document follows the Preferred Reporting Items for Systematic Reviews and Meta-Analyses (PRISMA). The PRISMA is a minimum list of things that must be reported in systematic reviews and meta-analyses. This list was made based on the evidence that was already available.

### Identification phase

The first step of current structured analysis of compositions was comprised of describing the publications that were examined. The current search analysis was restricted to the databases containing papers from conference proceedings and the documents considered the most important to the fields of AODV and relevant literature. Besides, broader searches of the SCOPUS and WoS database have been carried out to include related efforts from other origins not contained in the starting list. These databases include Scopus, Science Direct, Taylor Francis, The ACM Digital Library, Springer, IEEE, and Emerald Publishers. These seven databases that have the papers in professional journals and at international conferences are worth discussing, as shown in Table 1.

**Table 1:** Searched databases.

Data source	Website
SCOPUS	www.scopus.com
Taylor and Francis	www.tandfonline.com
THE ACM DIGITAL LIBRARY	dl.acm.org
IEEE	www.ieee.org
Springer	link.springer.com
Elsevier	www.elsevier.com
Science Direct	www.sciencedirect.com

The Systematic Review Database, Scopus, and Google Scholar were used to identify the modifications in the AODV routing discovery mechanism for wireless mesh network work already published in the open access publishing/online publishing and reviewed publications. Grey writings (*i.e*., the summary introduced at the conference), compositions, and incomplete articles were excluded from the analysis.

The database lists of all the studies were reviewed systematically. Analysts on the subject were classified as researchers and contributors. For each index, titles and abstracts of every publication have been inspected for keywords relevant to the mentioned issues: “AODV” Between 1900 and 2022, research papers written in English were found in the search.

### Criteria of screening

The screening phase assessed the articles at a deeper level to determine which articles listed could contain valuable material for the systematic literature review. Four researchers individually screened each item’s content during this process and marked it with a yes or no. Academic publications were extracted based on the above-listed search strategies. After eliminating duplicate papers containing redundant material, potentially significant articles were identified. Titles and abstracts were then scanned, and insignificant studies were excluded, leaving a total of potentially essential studies. Documents discarded were those that derived recommendations for specific applications (and were therefore not generalizable).

### Criteria of eligibility

Each article was assessed in-depth at the eligibility level. The researchers extracted the proposed recommendations and the specific studies conducted to validate or confirm those recommendations for each paper. The papers were eliminated from the current work if the suggested design guidelines contained design standards too broad for this work or too confusing for the researcher to understand (coders).

Several data processing tools were applied to keep records ordered during the identification and filtering phases. Online Google spreadsheets were used as a communication method. This made it possible for the researchers to work together. They could store and lookup records from the reviewed papers, analyze the data, and add notes.

### The phase of integration

Many researchers examined a qualitative analysis of the information extracted to better categorise the design guidelines and prepare the coded data to address the research questions that guided the systematic literature review. Although our study focused on a review of AODV studies published since the early 1900s, it nonetheless provides a snapshot of current research in this area. Based on this research, it is clear that several publications about WMN and *ad-hoc* changes to AODV routing discovery mechanics have been steadily going up since the turn of the century.

Inclusion and exclusion criteria were used to categorise relevant and non-related research, as all modifications to the AODV routing discovery mechanics in wireless mesh networks were included in the sample population. Furthermore, the current systematic review includes several recent articles about AODV modification in MNAET relevant to this review.

Inclusion requirements were based on a focused research topic as a prime precondition, modification to the AODV routing discovery mechanics in WMNs and execution while studying, a publication report available to the public or held between 1900 and 2022, recognisable rates of users, and promoted to all levels of users. A qualitative analysis concentrated uniquely on the abstracts of the papers, which did not fulfil any of the terms for implication and did not fall under the measures of exclusion. The current work was entirely checked for eligibility criteria to be calculated.

### Analysis of the methodological soundness of the studies/study’s qualitativeness evaluation

During the last phase of the procedure for collecting data, all of the necessary information was gathered, and any disagreements that existed amongst the authors were first discussed and then settled upon. Therefore, the authors summarised these articles. The process of interpreting and classifying the articles assisted us in obtaining a wide variety of essential and compelling hints. As a direct result of this, many potential future initiatives and recommendations have been proposed.

It is worth noting that the research investigation was conducted carefully, and it offered a detailed basis concerning modification to the AODV routing discovery mechanics in the wireless mesh networks. It is also imperative to keep in mind that the most challenging part of adopting the PRISMA methodology was coming up with indirect ways to describe the approaches in the abstracts and methodological parts of the publications.

Therefore, the authors had to go through the entire content of the papers and look at more knowledge to determine the particular approach used for changing the AODV routing discovery mechanics in assessing the requirements for wireless mesh networks. In this section, our literature review’s findings on our initial research questions have been discussed.

## Result

The current section represents the formal validity of the AODV. Also, the formal validity represents results in the form of a summary. Furthermore, the selection and extraction of articles will be outlined in the following subsection.

### Analysis of data sifting and extracting

A total of 627 papers were selected from over 1,000 for the initial search and identification process. The database lists of all the studies are reviewed systematically to do such work. A full-text review of the 175 articles was carried out to extract information from the correspondence in the eligibility process. This review omitted 50 more papers because they were too general or ambiguous to provide valuable research data on modifying the AODV routing discovery mechanics in wireless mesh networks.

This process resulted in 125 papers being marked as applicable to the AODV routing discovery mechanics in the wireless mesh networks for modification. In the end, in the course of the information withdrawal (‘Contained’ in the chart of PRISMA), the concluded set of articles was further checked to remove the related modification data found in the AODV routing discovery mechanics in WMNs and specifics of the studies that generated and validated the design guidelines. After carefully going through all the titles and abstracts, there were only 125 reviews that could be useful.

**Figure 8:**
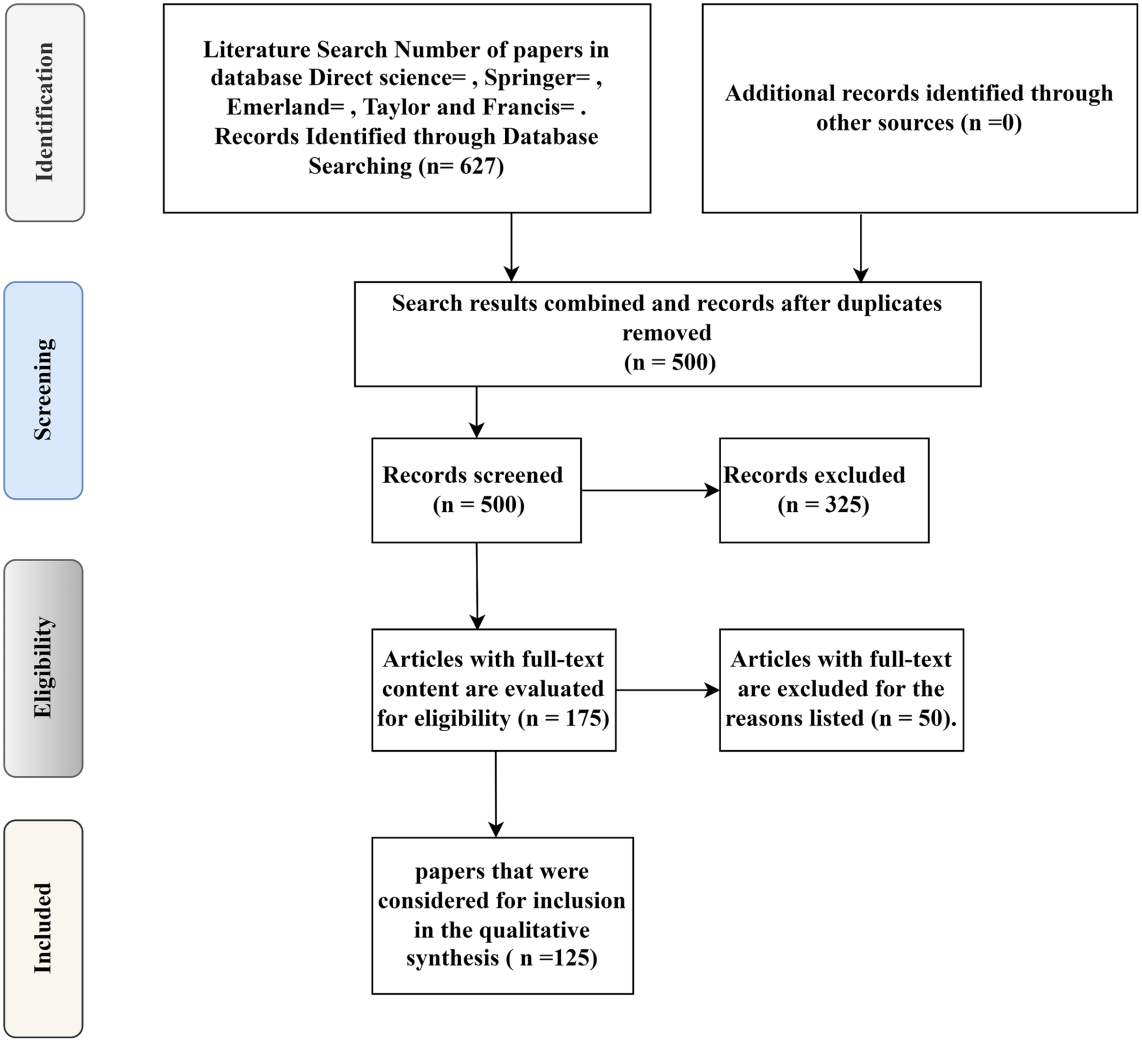
A flow diagram of PRISMA’s technique for selecting studies and extracting data.

In Fig. 8, the procedure used in current research for clarifying related papers can be followed in the diagram of PRISMA. Some of our previous work follows up on this interdisciplinary analysis of the literature in this area. However, the presented study focused on reviewing mobile learning meta-analytical studies published from the 1900s to 2022 to provide a snapshot of recent research. This analysis demonstrates that modifying the AODV routing discovery mechanics in wireless mesh networks is an evolving field of study, with a consistent rise in the number of publications since the early 1900s.

The data collected by the researchers was collected separately using the proper procedure and documentation. Interviews with the third analyst resolved disagreements, and a consensus was reached following the discussion. The required information was collected in the final stage of the data collection process, and any differences between the authors of this study were addressed and resolved. Then, the authors compiled the selected 125 articles. By translating and sorting papers, we were able to get a wide range of essential ideas.

As a consequence, many possible future initiatives and guidelines have been suggested. It should be noted that the study investigation was carefully carried out and provided a thorough basis for modification to the AODV routing discovery mechanics in wireless mesh networks. There was an issue with papers’ abstracts and methodological sections not explicitly expressing methods used during the PRISMA technique’s implementation. Therefore, the writers must go through the entire content of the articles and take a more in-depth look at more information to determine the exact form of the modification to the AODV routing discovery mechanics in the WMNs criteria assessment. This part of the selection process took a long time, but it ensured that the most relevant publications were chosen.

### Methodology for selection studies

The research group used personal feedback to develop and refine the paper selection criteria. The analysis was performed according to the standards established in the past analyses. Studies were categorised as either “important” or “not-important” based on the inclusion and exclusion criteria, such as informal literature (informal reports, ongoing development or work, technical notes, or PowerPoint slides). Also, as mentioned above, several papers have been left out because they were published in unknown indexes.

**Figure 9:**
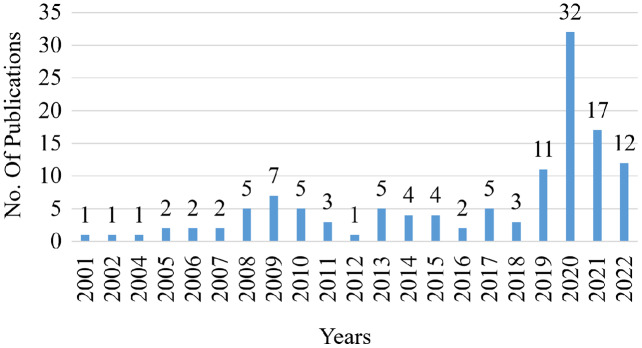
Selective paper publication by year.

The number of articles discussing AODV has increased dramatically in recent years, as shown in Fig. 9. Due to the expected delays in indexing and actual availability of published articles, the number of papers published in 2022 must be regarded as incomplete (as the query was achieved in June 2022). In addition, it is evident that the most significant number of publications ${({\rm N }=(11+32+17+12)72/125)}$ is increasing dramatically in 2019, 2020, 2021, and 2022.

### Study characteristics

The previous studies related to the modification of the AODV routing discovery mechanics in wireless mesh networks and MANET, which have been concluded from the last stage, have been summarised, and their characteristics are presented in Table 2. Based on the analysis conducted, it could be noted that some papers belonged to different countries. This indicates that the review has gathered information from the research conducted worldwide regarding the modification to the AODV routing discovery mechanics in WMNs. The included studies had different study designs (*e.g*., qualitative, quantitative, expository). More details are presented in Table 2 below.

**Table 2:** The features of the studies.

Authors/Years	Design of study	Strengths/Weaknesses
Jutao, Jingjing & Minglu (2009)	Experimental study	The suggested work aims to improve the QoS. The authors address the issues of packet delivery ratio and energy consumption, while the routing overhead and average daily still need improvements.
Makram, Samad & Gunes (2009)	Experimental study	This work was presented to support QoS in WMN. The result shows improvements in throughput and average end-to-end delay, while there is a gap in average packet delivery.
Kanakaris, Ndzi & Ovaliadis (2011)	Quantitative	Simulation results show that the presented protocol performs well in terms of data throughput and packet drop rate, while the energy consumption needs more investigation.
Piro et al. (2013)	Experimental study	The presented work needs more investigation regarding energy consumption because it is done in a small transmission range.
Chandel & Gupta (2014)	Comparative Analysis	The suggested work needs more research into the network parameters with virtual network challenges such as a higher number of nodes and energy consumption.
Tennekoon et al. (2014)	Experimental study	They proposed a hop-by-hop protocol that provided per-hop data encryption. The presented work needs more study to authenticate Service Providers of Record (SoRs), clients, and apps.
van Glabbeek et al. (2016)	Experimental study	This paper presented a new version of AODV using AWN (Algebra for Wireless Networks). The supposed work needs study because several points are not addressed in the work.
Tarapiah, Aziz & Atalla (2017)	Experimental study	Authors discussed measuring tools for two mobility models’ protocols. This model utilises NS-2 VANET simulators.
Zugno et al. (2019)	Experimental study	The authors reported the simulation of cellular networks with NS-3 and its models.
Li & Peng (2020)	Experimental study	The work was applied with a multi-path routing protocol and needed to be examined with a single-path rather than multiple paths as previously proposed.
Haglan et al. (2020)	Quantitative	Based on the simulation findings, it is apparent that increasing the number of nodes in the network or the network’s size will negatively impact network performance.
Farheen & Jain (2020)	Experimental study	The suggested routing protocol suffers from network overhead with a loading number of nodes. NS-2 simulates the proposed work.
Mostafa et al. (2020)	Experimental study	The authors suggested enhancing the routing protocol due to the unacceptable result in terms of E2E delay—the study conducted by NS-2.
Campanile et al. (2020)	Systematic Review	The authors suggested an investigation with other tools, such as NS-2 and OMNET ++, for the scientific community and practitioners to identify what is currently not covered and propose targeted built-in expansions.
Bamhdi (2020)	Experimental study	The proposed work has an issue regarding overhead that various node densities will increase with the increasing number of nodes.
Al-Bayram & Abdullah (2021)	Quantitative	The study concluded that the routing protocol they used in their study could be proper for the small area of networks but not for large coverage areas.

### The AOVD’s routing discovery mechanics modifications in wireless mesh networks

The *ad-hoc* on-demand distance vector (AODV) protocol is the widely used hop count protocol, which operates on need and utilises an efficient route discovery mechanism. The route from source to destination is usually determined by the route discovery mechanism, where the address of each node is traversed to reach its destination. It is a common routing protocol to create the topology of a network. It responds quickly to dynamic link conditions, low processing and memory overhead, and low network utilisation and determines uni-cast routes to destinations in the *ad-hoc* network. It uses destination sequence numbers to prevent a loop and thus prevents sending the same packet to the node from another node.

A standard method of AODV routing transmits the route request to all nodes (Kanakaris, Ndzi & Ovaliadis, 2011). Analysis results in a suggested action plan and provides a list of nodes in a provided zone (one-hop nodes). Only a set of nodes in each community is allowed to be transmitted when a message is sent. Depending on the requirement and the appropriate service quality, the number of the selected nodes may be varied dynamically. At the same time, each network interface will forward a route discovery process message if a state dependent on its neighbour concentration is resolved in that case. Due to redundant delivery, the proposed scheme minimises network congestion.

Only one possible route includes the node if the RREQ is obtained from an intermediate node in its route list. These mentioned nodes form part of this route list if the chosen nodes can only transfer the route request packet. The intermediate node’s neighbourhood density is regarded in this proposed scheme as the factor in the forwarding plane of RREQ for the in-between node. This implies that the possibility of transmission of any node will become low if the neighbour’s count of nodes increases. Finally, the above information will decrease. The picking of nodes improves the likelihood of complete network coverage at random from the neighbourhood set. More significant savings could be achieved by choosing nodes for transmission using a range-dependent strategy. This can only be done at the expense of expanding the scope (Kanakaris, Ndzi & Ovaliadis, 2011).

The protocol, AODV, is for reactive routing based on route exploration and path conservation protocols. The path discovery process is established only when a node has a package to deliver. A node that wishes to transmit packets creates RREQ packets and broadcasts them when it finds a path. For this purpose, modifications are required to the AODV protocol relating to the route discovery procedure (Bugarcic, Malnar & Jevtic, 2019).

As a reactive algorithm, AODV has the advantage of not needing to keep track of routes for nodes that are not actively communicating. Bellman-Ford’s “counting to infinity” problem is stopped by AODV, which tells the affected nodes about the status of the current link. Employing sequence numbers per request helps the nodes execute the route request they have already processed. The multi-cast group’s IP address should be set as the destination node for the multi-cast route. After the route is discovered, the source node must uni-cast a Multi-cast Activation (MACT) message to activate the route (Belding-Royer & Perkins, 2002). The advantage of AODV is that it needs fewer resources than OLSR because the control messages and the routing tables of AODV are small. OLSR continuously consumes bandwidth with topology update messages. This makes AODV a better option for restricted bandwidth and computational resource systems. On the other hand, OLSR performs better in an extensive network with dense traffic (Johnson, 2009).

### Simulation-based routing protocol analysis

The reported simulated routing protocols have been analysed by Hu, Pirzada & Portmann (2006). A pair of tests were performed to assess the AODV protocol’s performance under changing mobility and traffic loads. The mesh mobility in the first test ranges from 0 to 20 m/s and keeps the transmission rate set at 512 kbps. The transmission rate rises from 256 kbps to 4 Mbps in the second test while maintaining the overall mesh client speeds at 5 m/s. For the first test, the results are shown in Fig. 5. The findings obtained using NS2 are labelled AODV-NS2, and those obtained using MNE from the testbed are labelled AODV-MNE.

**Figure 10:**
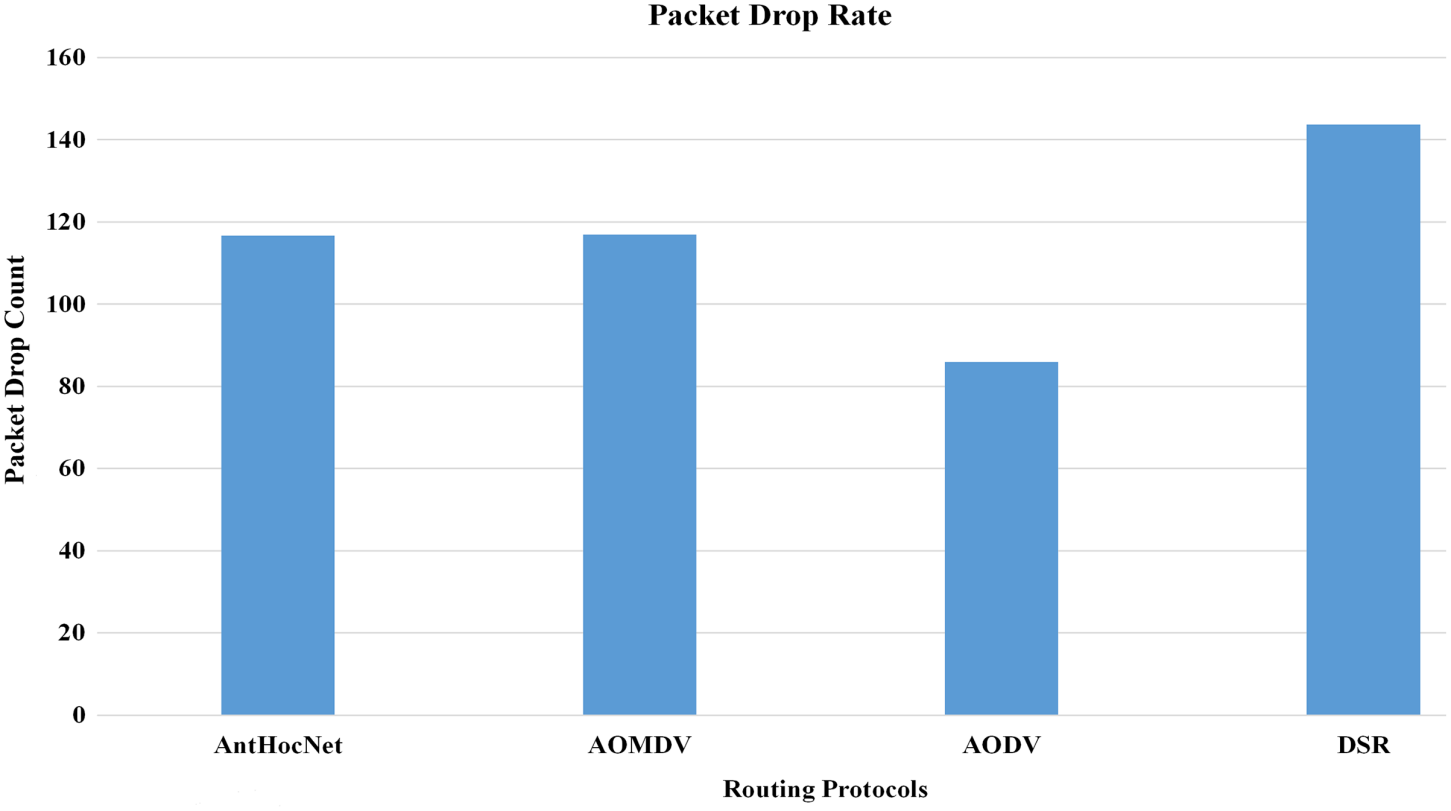
Routing protocols evaluation (Alameri & Komarkova, 2022).

**Figure 11:**
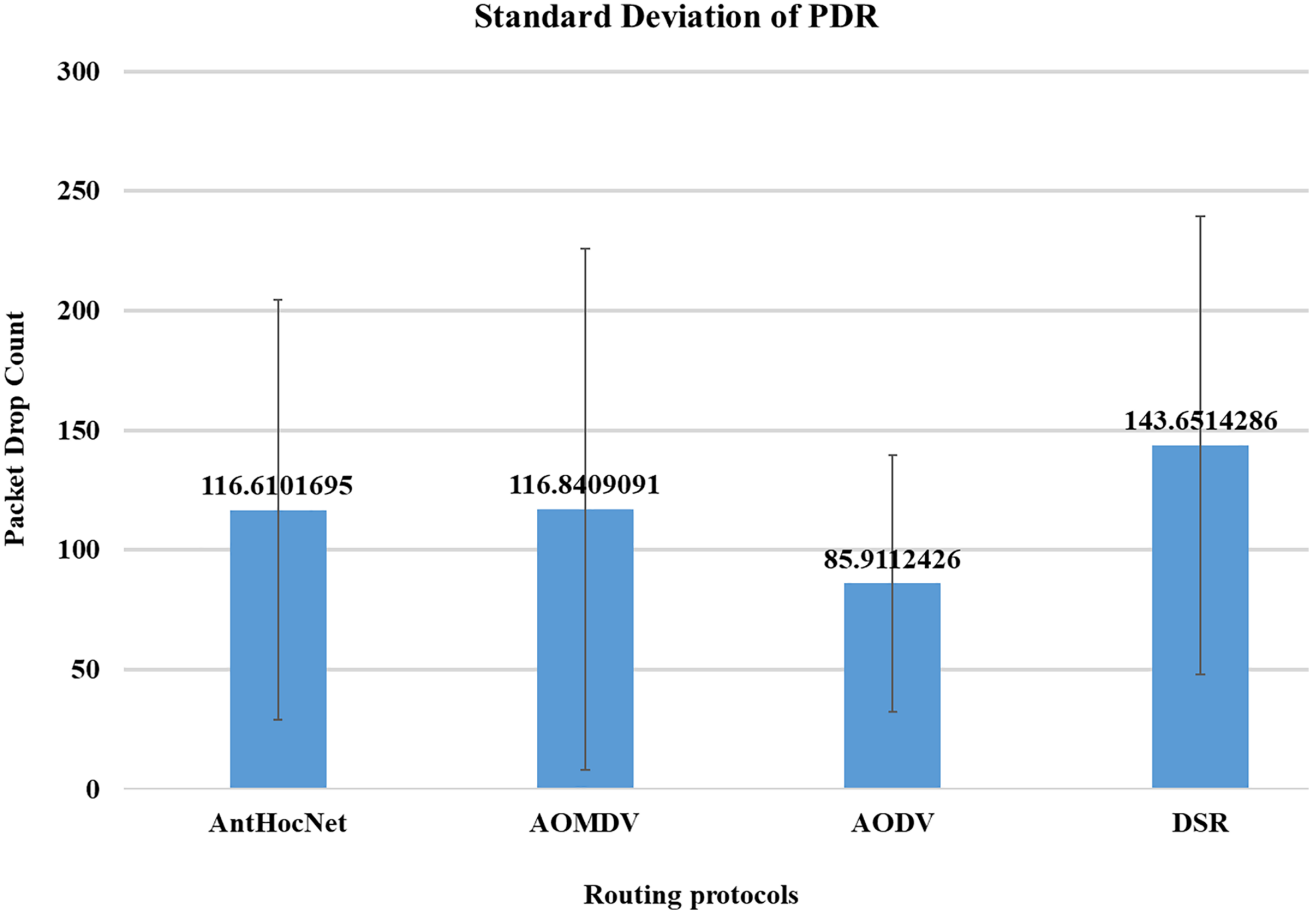
Routing protocols evaluation (Alameri & Komarkova, 2022).

**Figure 12:**
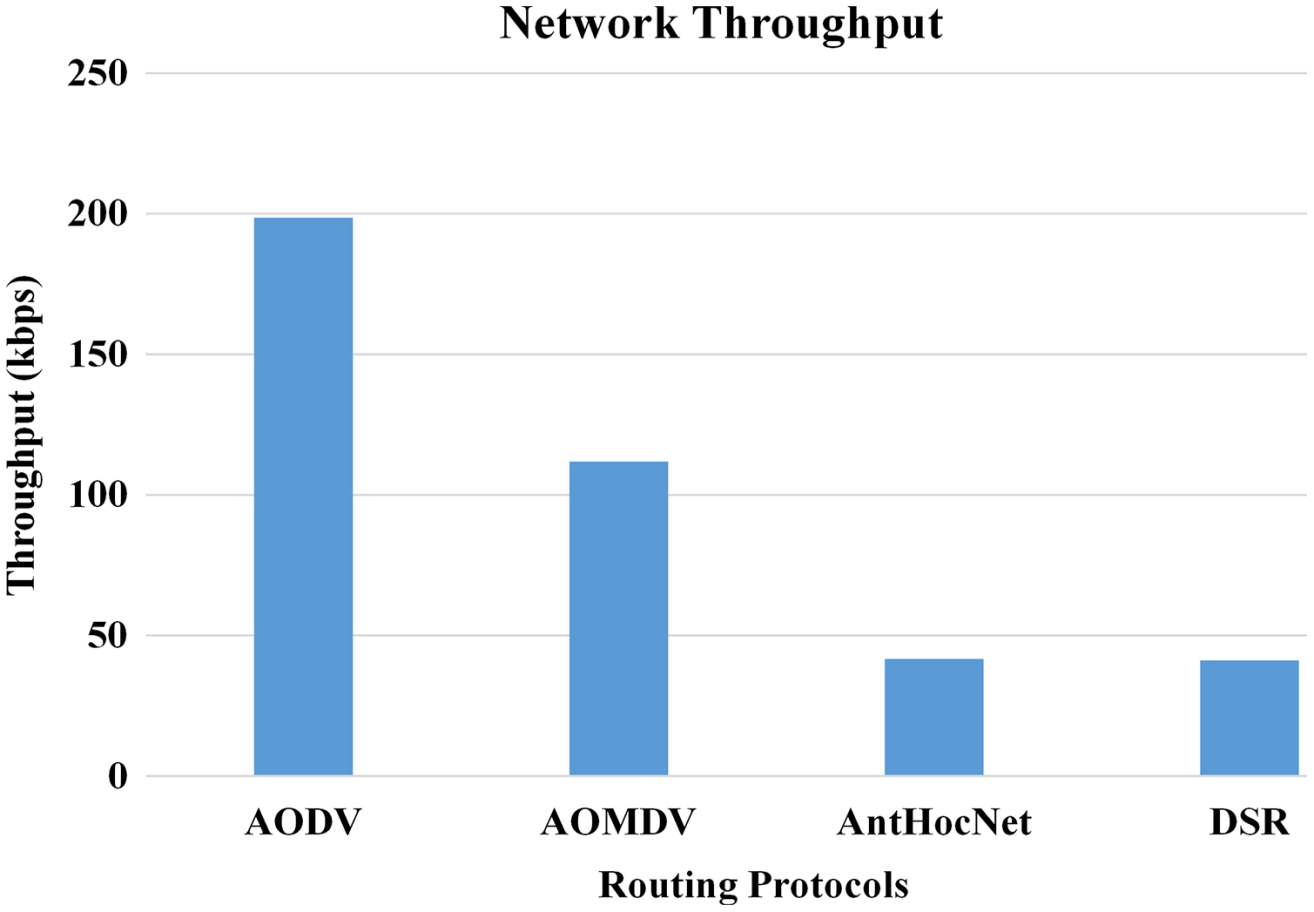
Routing protocols evaluation (Alameri & Komarkova, 2022).

**Figure 13:**
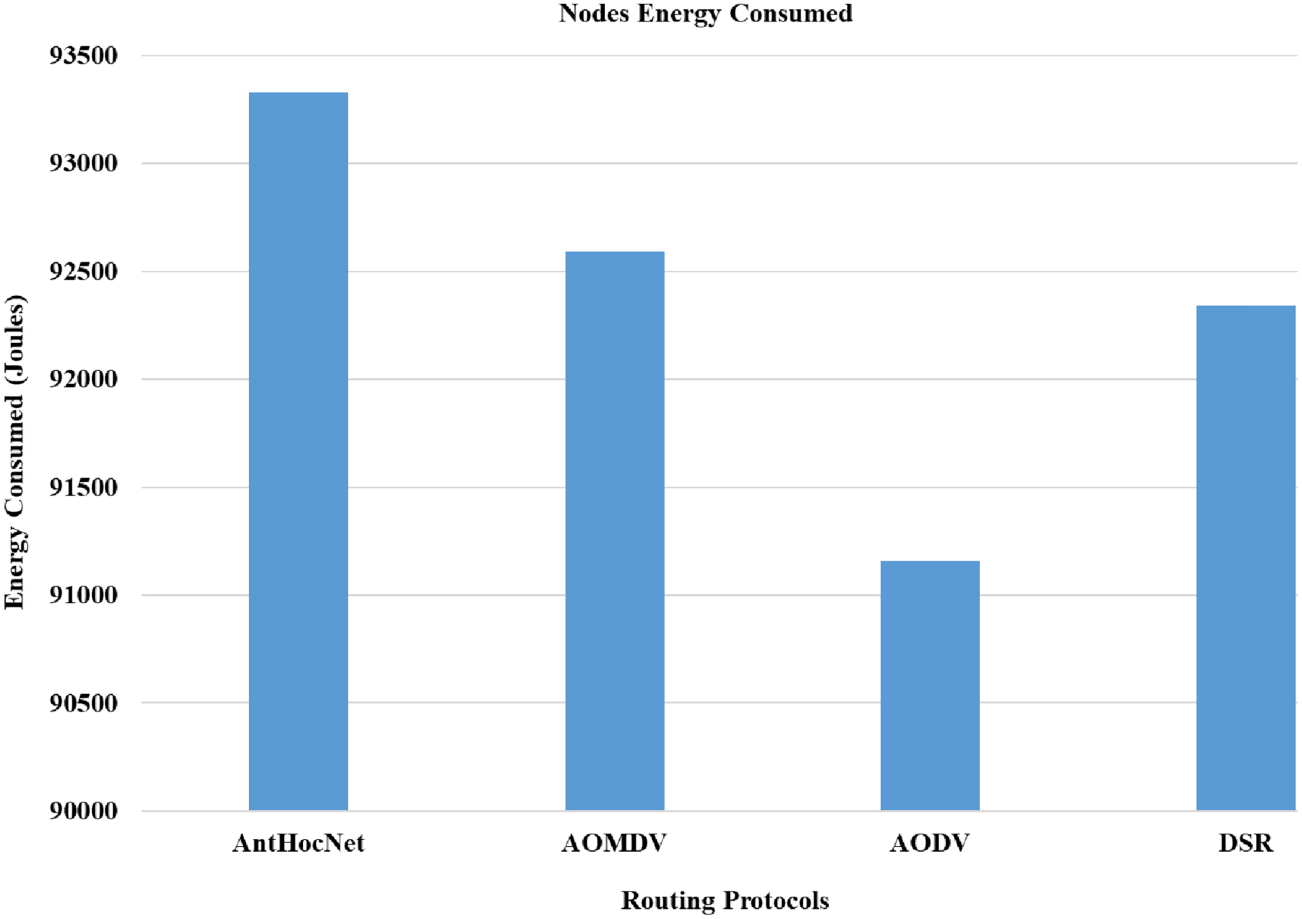
Routing protocols evaluation (Alameri & Komarkova, 2022).

In the proposed work of Alameri & Komarkova (2022), the authors evaluated the effectiveness of various routing protocols in MANET using the NS-2 simulator. As part of the evaluation, mathematical statistics have been used to study and evaluate the behaviour of the transmitted data and measure the efficiency of the protocol based on the simulation results and the standard deviation of the data. In this work, they used several parameters for performance evaluation that are used in this study, such as packet drop rate (PDR), throughput, and energy consumption. The AODV routing protocol performs better in terms of PDR, network throughput, and energy consumption. Figures 10–13, shows more details about the routing protocol parameters.

Although some studies (Suhaimi, Mamat & Azzuhri, 2010), have suggested new route recovery schemes for the algorithm of AODV regarding the issue of crushed ties, a relative comparison in the middle of the manageable effect on the varying degrees of flexibility, dealing burden, and other source-station sets and the process earlier introduced in the primary AODV Request For Comments was not performed at all. The part below describes the AODV request local recovery process executed in the reported reports, presentation appraisal contrast, and findings. Suhaimi, Mamat & Azzuhri (2010) investigated the efficiency of AODV in connection failure situations due to node mobility and the consequent mechanism of route recovery using NS-2 simulators.

The relationship between the destination node and the source ties of the three pacts and the number of nodes in the region has also been shown in Li & Peng (2020). The article suggested the Fortified Ant protocol’s performance parameters can be substantially better than the standard DSR protocol and the Ant colony algorithm (ACO). It has been demonstrated that the ACO-based sorting addition can efficiently speed up the mesh network in Li & Peng (2020).

In Li & Peng (2020), the authors depicted the three configurations’ average communication delay and network quantity. When the number of nodes in the area is set in this simulation, a different number of nodes with source and destination addresses is randomly generated, the number of connections in the mesh network is increased, and then the communication delays simulated by the three systems are compared, and the reinforced network is compared. The simulation impact of the Ant protocol is more substantive. Finally, the three protocols’ sufficient transmission power is simulated under different link numbers, simulation results are obtained. It can be shown that the multi-path transmission protocol’s success rate of transmission is higher than that of any given network propagation. The Fortified Ant protocol is also flourishing in this simulation. It seems likely that the Reinforced Ant protocol can be made to work better in a Mesh network.

The experimental results show that the Fortified Ant protocol’s connection search speed is remarkably higher than the conventional ACO. The protocol’s norm is end-to-end postponements, and the transference performance fare is better recommended than the two AODV and AOMDV networks. The simulation aims to examine the impact of increasing the network size, end-to-end delay, and packet delivery ratio on different VANET environment routing protocols (AODV, DSR, DSDV, AOMDV) using the NS-2 simulator.

More attention has focused on the provision of AODV with his suggested work of Bamhdi (2020). The paper states that the two-ray and freeway mobility models are simulated using the same parameters at different node densities (75, 100, 150, 200). The analysis provides conclusive proof of the benefits of DP-AODV over standard AODV, as shown by the study’s findings. Due to the use of single path transmission by both routing protocols, the analytical comparison of results is more realistic and accurate. Files simulating various traffic and movement patterns were run with both routing techniques to eliminate bias.

It can be shown that the packet delivery fraction has continuously improved in all kinds of densities with the use of DP-AODV compared to normal AODV. The proposed technology limits density by managing transmission power, which accounts for the increase from 12% to 31%. The dropped packet rate is higher in basic AODV than in DP-AODV, and many packets are lost due to fixed transmission power, producing extensive interference between nodes. Also, the delay has decreased with DP-AODV for most densities. Currently, it is at 52%, down from 54%. Therefore, DP-AODV is typically inferior to standard AODV when looking for routes with a higher success probability and lower data transmission latency.

In contrast to AODV, where many packets are lost due to fixed transmission power and severe interference between nodes, the proposed DP-AODV limits density by regulating the power used for transmission. The author presented the results obtained from the experimental studies. The DP-AODV was found to be better in both terms of throughput and jitter. The suggested protocol becomes more acceptable and produces high performance in terms of throughput and jitter network parameters.

The DP-AODV is applied to the single-path transmission in the study scenarios. However, the study still lacks knowledge of the behaviour of the new DP-AODV with the multi-path transmission. Furthermore, the DP-AODV race has an overhead as the number of nodes in a network grows. It is a noticeable overhead that various node densities will increase with the increasing number of nodes. The author compares overhead with various nodes in Bamhdi (2020).

The comparison was carried out with simulation tools (*e.g*., NS3 and NS2). In already documented studies, different mobility models were considered to construct the network scenario more like the real world. Studying various routing protocols provided us with the experience of using the correct protocol under different network conditions in multiple instances.

## Critical analysis

It is critically assessed that *ad-hoc* vehicular networks’ unique characteristics, such as high node speed and frequent topology changes, pose challenges to the routing process. The advent of the Internet of Vehicles (IoV) definition and autonomous and connected vehicles leads to new innovative technologies with various service quality requirements, thus increasing the new challenging data transfer problems. So far, several routing protocols have been proposed for such connections. *Ad-hoc* on-demand Distance Vector (AODV), Dynamic Source Routing protocol (DSR), Optimum Link State Routing (OLSR), and Destination-Sequenced Distance-Vector Routing Protocol (DSDV) are among the most common ones. This analysis tests the routing mechanisms of AOVD for WMNs and their modifications.

The analysis starts with the implementation of WMN architectures and routing function basics. Thanks to their most significant pledge to incorporate various descendants’ wireless networks, the WMNs have been the subject of research in recent years. Modern communication systems have been the subject of recent studies on WMNs, motivated by rich and high-speed content access. In contrast, the suggested protocols have comparatively received little attention. There is a broader field of applications. Effective routing protocols may offer significant advantages in efficiency and reliability for mobile *ad-hoc* networks. AODV is ideal for networks with modest computational power and relatively light traffic as a reactive protocol. The advantage of AODV is that it does not create overhead for inactive nodes, which turns out to be a disadvantage as the traffic load increases. The lack of network topology information causes AODV to under-perform. Reviewed literature shows that conventional AOVD was slow in performance and information changed with time, and conventional could not get that information appropriately. So, we have conducted a literature review to highlight the modification in the AOVD routing protocol in wireless mesh networks.

Previous studies also noted that AODV had been modified for transmitting route request messages. The updated version was called AODV EXT. Unlike possibility-based strategies, where a fixed probability can be allocated to each node that does not guarantee complete network coverage, the methodology suggested in previous studies and this paper review incorporates eliminating repetitive re-transmissions on routing paths, principles from complete range routing algorithms with node pruning. Still, it provides interconnection and is more robust in the route path than wireless network guarantees.

The findings of the present study can also be compared to the routing protocol proposed by Khelifa & Maaza (2010), for the ER-AODV (*i.e*., Energy Reversed *ad-hoc* On-Demand Distance Vector, which contains up to 1.7% more power in comparison with that of *ad-hoc* On-Demand Distance Vector Request EXT). Compared to the standard AODV, AODV EXT increases data throughput by more than 19% and 10% more than the ER-AODV.

The conclusions Khelifa & Maaza (2010), also indicate that constructive agreement has low performance on large networks, although they are more effective in communication. On the other hand, reactive protocols have better performance on extensive network connections. Because of high expenses related to the path and that of the nodes’ shift, dual classes of the protocols execute poorly on immense mobile networks. Compared to standard protocols, a composite protocol (*i.e*., AODV) demands a solution and has previously suggested eliminating inefficient re-transmissions depending on the transmitted node. Neighbourhood densities have shown pledging outcomes. This research has shown that for *ad-hoc* networks to be as efficient as possible, protocols would need to be fine-tuned to fit different traffic or applications.

## Conclusion

The authors conducted a systematic literature review identifying research patterns on AODV routing discovery dynamics modification in wireless mesh networks. This research review article concludes that modifications to the AOVD routing protocol have been presented. For this highly mobile network, the AODV protocol is proposed, but AODV only saves a minor hop route to the destination. The path that increases the network overload needs to be rediscovered when the connection breaks. In this review paper, the researchers highlighted the traditional AODV previously invented and addressed a new addition to the AODV (AODV routing protocol) that inspects path strength to create a more secure route between the destination and source. Studies show improvements in the format of RREQ and Hello messages to track the time of sending and path stability in the new versions of AODV. A comparison and review of previously published simulation results were performed to determine the efficacy of the AODV. It has been found that the AODV shows improved efficiency in several aspects.

The study presents an outline of all the essential aspects of AODV, which will be helpful for academics working in this field who are looking into this topic. In conclusion, based on this survey, the current routing protocol mentioned above, AODV, has been extensively researched. However, there are still ways to improve the protocol to ensure quality in an unreliable mobile *ad-hoc* network with few resources in future work.
